# The effect of misophonia on cognitive and social judgments

**DOI:** 10.1371/journal.pone.0299698

**Published:** 2024-05-09

**Authors:** Heather A. Hansen, Andrew B. Leber, Zeynep M. Saygin

**Affiliations:** Department of Psychology, The Ohio State University, Columbus, OH, United States of America; Harvard Medical School, UNITED STATES

## Abstract

Misophonia, a heightened aversion to certain sounds, turns common cognitive and social exercises (e.g., paying attention during a lecture near a pen-clicking classmate, coexisting at the dinner table with a food-chomping relative) into challenging endeavors. How does exposure to triggering sounds impact cognitive and social judgments? We investigated this question in a sample of 65 participants (26 misophonia, 39 control) from the general population. In Phase 1, participants saw faces paired with auditory stimuli while completing a gender judgment task, then reported sound discomfort and identification. In Phase 2, participants saw these same faces with novel ones and reported face likeability and memory. For both oral and non-oral triggers, misophonic participants gave higher discomfort ratings than controls did–especially when identification was correct–and performed slower on the gender judgment. Misophonic participants rated lower likeability than controls did for faces they remembered with high discomfort sounds, and face memory was worse overall for faces originally paired with high discomfort sounds. Altogether, these results suggest that misophonic individuals show impairments on social and cognitive judgments if they must endure discomforting sounds. This experiment helps us better understand the day-to-day impact of misophonia and encourages usage of individualized triggers in future studies.

## Introduction

Misophonia is a recently defined condition characterized by a strong aversion to certain background noises. While much has been done to categorize the prevalence of the condition–studies surveying symptomology in the U.S., U.K., and China have found moderate to severe misophonia symptoms in up to 20% of their populations [1–3]–most knowledge of impairment associated with misophonia has been collected via self-report. For instance, individuals with misophonia have described trouble focusing (e.g., during a movie or lecture [4]) when trigger sounds are present, and large-scale surveys of misophonia consistently report moderate or severe interference in social or work life [1,5]. Indeed, the recent consensus definition of misophonia recognizes impairment in social, occupational, or academic functioning as a marker of misophonia [6].

Humans are undeniably social beings; social interaction is crucial for health and survival [7]. However, there has not been experimental investigation thus far into the social effects of misophonia. We know from large-scale self-reports that individuals with misophonia commonly externalize their reactions to the poor behavior of others [4,8]. For instance, participants commonly find a trigger-producing person “rude” or feel negative emotions toward them such as “I hate this person” [4]. In a study prompting participants to identify presented sounds, some misophonic individuals defaulted to external judgments and social blame, writing things like “chewing pig person” or “crunching cave person with no manners” for the sound of chewing [9]. Does misophonia systematically alter how others are viewed?

The impact of misophonia on cognitive functioning has received a bit more academic investigation. Experimentally, individuals with misophonia have shown decreased cognitive performance in the presence of triggers. For example, a confederate chewing gum next to participants reading a paragraph impaired paragraph retention for sound sensitive participants on a subsequent comprehension test [10]. Other work has shown deficits in selective attention [11,12] and cognitive control [13,14] in the presence of oral/nasal triggers for individuals who experience misophonic sensitivity. These prior studies have concluded that individuals with misophonia demonstrate impairment in the ability to retain relevant information or withhold responses to irrelevant distractor stimuli.

While the cognitive consequences of misophonia have been investigated in a handful of studies, this research will go further in the following ways. First, prior work has demonstrated cognitive impairment in the presence of oral/nasal stimuli, but individuals with misophonia experience aversion to more than just oral/nasal sounds [1,6,9]. For instance, misophonic participants rate both human-produced non-oral/nasal sounds (e.g., typing, clicking a pen) and nonhuman/nature sounds (e.g., water dripping, dog drinking) as evoking more discomfort than controls do [9], and non-oral/nasal (i.e., finger) sensorimotor brain regions show connectivity differences in individuals with mild misophonia compared to controls [15]. Is cognitive impairment exclusive to oral/nasal sounds, or will non-oral sounds likewise impair performance? Similarly, most misophonia research thus far that has presented auditory stimuli has utilized a single instance of each trigger sound, such as one representation of gum chewing (e.g., [9–11,14,16–19]). However, not all individuals with misophonia are bothered by the same stimuli; for instance, some individuals are only bothered by a loved one chewing rather than a stranger chewing [4,20], so a single exemplar might not capture that individual’s aversion. The use of multiple exemplars of a wide variety of sound categories would therefore be a useful next step.

To quantify social and cognitive judgments, we turn to face memory paradigms used in literature on trait formation and subsequent memory. For instance, when participants are shown faces paired with either positive or negative behavioral descriptors in a learning phase, they subsequently rate faces paired with positive descriptors more positively and faces paired with negative descriptors more negatively [21]. Moreover, source memory for faces associated with negative descriptors (i.e., cheating) is better than that for faces associated with positive or irrelevant descriptors (i.e., trustworthiness) [22]. While face memory (i.e., old-new discrimination) did not differ by description in [22], other work using stimuli with emotional valence (e.g., fearful vs. neutral faces) has demonstrated a memory enhancement for these emotional stimuli (see [23] for a review). In addition to visual information, complex auditory distraction during learning has also been shown to alter performance. Compared to faces presented with steady-state sequences, faces presented with changing-state sequences are remembered less often in a subsequent memory task, and both types of auditory distraction impair face memory relative to quiet [24]. Can we impact trait ratings or memory performance by pairing faces with triggering or non-triggering sounds?

The main aims of the present manuscript are to explore whether exposure to oral and non-oral trigger sounds alters cognitive or social judgments in misophonia. To address these questions, we utilized an incidental memory encoding paradigm where participants viewed faces paired with sounds, reported the gender of the face, then identified the sound and rated their experienced discomfort (Phase 1). After, participants were given a surprise memory test of these (and novel) faces where they reported how much they liked the face, whether they remembered seeing the face, and which sound (if any) was initially introduced with the face (Phase 2).

We have four specific questions: 1) Can we replicate prior work showing misophonic discomfort to non-oral trigger sounds? We predicted that individuals with misophonia would rate oral and non-oral trigger sounds as evoking more discomfort than controls do, but groups would show no difference in discomfort to control sounds; further, we predicted that oral and non-oral trigger sounds that were correctly identified would elicit more discomfort in misophonia than sounds that were incorrectly identified. 2) Do trigger sounds affect accuracy or response time on a task simultaneous with sound presentation? We sought to compare responses both within-group (e.g., task performance during trigger vs. non-trigger sounds within the misophonic group) and between-group (e.g., task performance during trigger sounds in misophonic participants compared to controls). We predicted that individuals with misophonia would make more errors on a concurrent gender judgment task when listening to trigger sounds, compared to a) when listening to non-trigger sounds, or b) control participants listening to trigger sounds. 3) Do trigger sounds influence subjective trait judgments of otherwise neutral face stimuli? Using similar within-group and between-group analyses as Aim 2, we predicted that individuals with misophonia would rate faces paired with trigger sounds as less likeable than a) faces paired with non-trigger sounds or b) how control participants rate faces paired with trigger sounds. 4) Do trigger sounds presented during face learning influence memory performance on a surprise memory test? We predicted that memory for the face and/or sound will differ between individuals with misophonia and controls, either showing decreased memory performance (e.g., from distraction) or increased memory performance (e.g., from an emotional enhancement of memory).

## Method

### Surveys

#### Duke-Vanderbilt Misophonia Screening Questionnaire (DVMSQ)

Two surveys were used to assess misophonia level. The DVMSQ [25] was chosen for its brevity, clinical cutoffs and research utility. The survey includes a screening item (“*Are there specific sounds that you are extremely bothered by*, *even if they are not loud*? *Yes/No”)* and 18 follow-up questions on a 5-point scale for participants who select “Yes”. The follow-up questions probe symptom frequency (score range: 0–40) and interference/impairment (score range: 0–28) for a total score range of 0–68, where higher scores indicate more severe misophonia. The DVMSQ offers theory-based diagnostic criteria dependent upon responses to the follow-up questions; if respondents meet all symptom criteria with impairment in daily functioning they are considered to have “clinically significant misophonia”, whereas if they meet all symptom criteria except for impairment they are considered to have “sub-clinical misophonia”. For a breakdown of participant responses on the DVMSQ subscales and resultant diagnostic labels, see S1 Table in S1 File. Of note, one criterion mandates a loss of self-control or urge to be violent following trigger exposure, which is a rare symptom [6]; we found this to be the disqualifying factor for 7 of our 65 participants between having misophonia and not. As such, S1 Table in S1 File likewise contains “adjusted” DVMSQ labels removing this criterion, with labels as follows: 1) clinically significant (meeting all remaining criteria), 2) sub-clinical (meeting all remaining criteria except for impairment), 3) mild (meets some criteria), and 4) none (answered “no” to the screening question or answered “yes” but meets no criteria). We provide this information as a thought exercise for future application of this questionnaire.

#### Selective sound sensitivity syndrome scale (S5)

Additionally, the S5 [8] was chosen because of its ability to capture subfactors of misophonia experiences (externalizing, internalizing, impact, outburst, and threat) and its successful usage in multiple languages, including English (U.K.), German, and Mandarin [26–28]. The S5 contains five questions for each of the five subfactors, e.g., externalizing: “*People should not make certain sounds*, *even if they do not know about others’ sensitivities*”; internalizing: “*The way I react to certain sounds makes me wonder whether deep inside I am just a bad person*”; impact: “*My job opportunities are limited because of my reaction to certain noises*”; outburst: “*I can get so angry at certain noises that I get physically aggressive towards people to make them stop*”; threat: “*I feel trapped if I cannot get away from certain noises*”. Each question is presented with a 10-point response scale, with higher responses indicating more severe experiences. It has a recommended cutoff score of 87 out of 250 to classify misophonia [28]. For further information about the psychometric properties and validation of the DVMSQ and S5, we encourage readers to consult the respective manuscripts.

Lastly, to account for performance differences that may be due to other psychiatric disorders, the Obsessive Compulsive Inventory-Revised [OCI-R; 29] and Depression Anxiety Stress Scale-21 [DASS-21; 30] were also given to participants.

### Participants

We used *a priori* power analyses from pilot data (with the S5 to determine misophonia level, since the DVMSQ was not yet available) to determine the sample size for this experiment, assuming unequal group sizes since fewer individuals presented with misophonia than without in our pilot. Calculations determined that a sample of 21 individuals with misophonia and 31 controls is sufficient to achieve a difference in discomfort ratings to misophonic trigger sounds (effect size: *d* = 1.047) with 95% power, and a sample of 24 individuals with misophonia and 36 controls is sufficient to achieve a difference in trait ratings (effect size: *d* = 0.981) with 95% power. As such, we collected data until we obtained at least 24 individuals with misophonia and 36 controls, as determined by the S5 questionnaire.

In total, 69 participants were recruited to participate in this study in the Fall of 2022. 39 participants were undergraduate students who were enrolled in an Introduction to Psychology course at The Ohio State University and received course credit for their participation. 30 participants were members of the community recruited via flyers, Reddit, and word of mouth who received $10 for their participation.

Of the 69 participants, three did not finish the experiment due to computer error. Additionally, due to known differences in memory performance across the lifespan [e.g., 31] participants older than 50 (N = 1) were not analyzed and excluded post hoc. This led to a final sample of 65 participants (Mean Age = 20.8, Range = 18–37; 37 Females, 22 Males, 6 Nonbinary/Other).

#### Misophonia assessments

*S5 groups*. Using *a priori* group divisions based on the S5, these 65 participants broke down into 26 individuals with misophonia (Mean Age = 22.1, Range = 18–37; 20 Females, 2 Male, 4 Nonbinary/Other) and 39 controls (Mean Age = 19.9, Range = 18–34; 17 Females, 20 Males, 2 Nonbinary/Other). The misophonia group had an average S5 score of 135.7 (Range = 92–207), and the control group had an average S5 score of 21.6 (Range = 0–69). Of note, the control group was not gender matched to the misophonia group and was significantly younger (*t*(63) = 2.033, *p* = 0.046).

**Fig 1:**
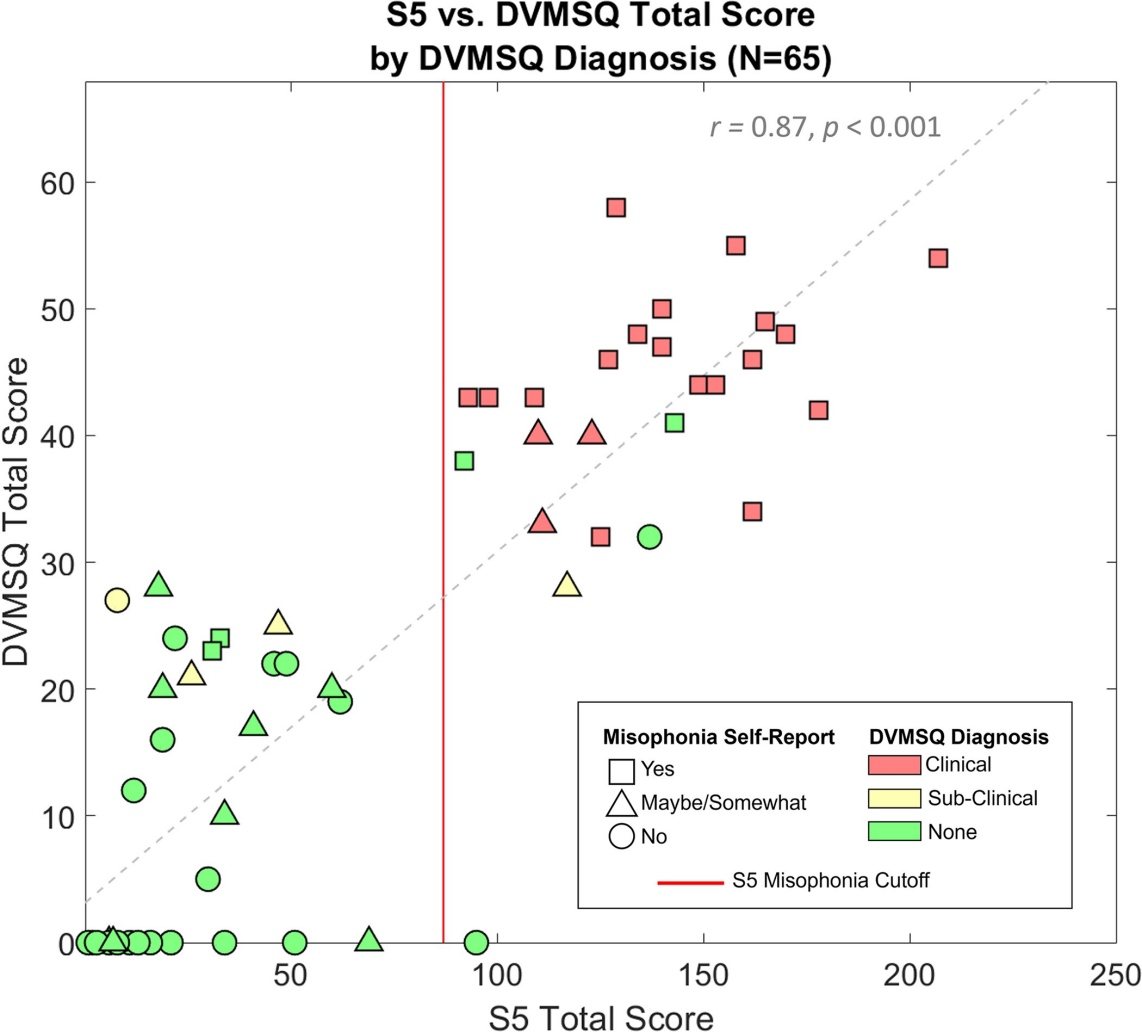
Misophonia assessment comparison. S5 total score (out of 250) by DVMSQ total score (out of 68), split by participant response to “Do you think you have misophonia?” (shape) and DVMSQ diagnostic assessment (color). Red vertical line (x = 87) separates individuals with and without misophonia, according to the S5 total score.

*Extreme groups*. Given that the DVSMQ and S5 have not yet been used in conjunction with one another, we found it useful to compare responses across the two surveys. We compare these survey metrics against a single self-identification question (following experimental debriefing and an explanation of misophonia) that read *“Do you think you have misophonia*?*”* and included options of *“Yes”*, *“Maybe/somewhat”*, and “*No*”. For a depiction of how participants responded, see Fig 1.

While the S5 and DVMSQ total scores are highly correlated (*r* = 0.87, *p* < 0.001), the DVMSQ diagnoses and self-report responses revealed more mixed results. For example, we observed an individual self-identifying as not having misophonia and answering “No” to the screening question of the DVMSQ, but scoring higher than 87 on the S5 and thus falling into the misophonia group; in contrast, we observed another individual self-identifying as having misophonia and meeting all the symptom criteria of the DVMSQ (sans impairment), but scoring lower than 87 on the S5 and therefore landing as a control. Given the variability in responses across the three metrics and a lack of recommendation thus far on a particular survey to use, for analyses in which group comparisons are useful we will report both A) group divisions using the S5 only as chosen *a priori*, and B) “extreme” group divisions using a post hoc conservative metric. For these extreme group divisions, comparable to the method used in [18], individuals with misophonia were classified as any participant who 1) self-reported “Yes” to having misophonia, 2) met the criteria for clinically significant misophonia by the DVMSQ, and 3) scored above 87 on the S5. Conversely, participants were considered controls if they 1) self-reported “No” to having misophonia, 2) answered “No” to the screening question of the DVMSQ and/or met none of the symptom criteria, and 3) scored below 87 on the S5. Using this method, the extreme misophonia group included 18 participants (Mean Age = 23.0, Range = 18–37; 13 Females, 1 Male, 4 Nonbinary/Other) with an average S5 score of 144.4 (Range = 93–207). The extreme control group included 26 participants (Mean Age = 19.8, Range = 18–34; 9 Females, 16 Males, 1 Nonbinary/Other) and had an average S5 score of 17.1 (Range = 0–62). Of note, the control group was not gender matched to the misophonia group and was significantly younger (*t*(42) = 2.220, *p* = 0.032).

For a table of demographic information and assessment scores for each participant, see S1 Table in S1 File.

### Stimuli

#### Faces

The face stimuli used in this study came from the Chicago Face Database, combining both the main release [32] and multiracial expansion [33]. Each face was depicted from the shoulders up against a white background, with individuals donning a gray shirt and neutral expression. Piercings and facial hair were digitally removed, and final stimuli were equated in size and color temperature.

We specifically sought faces with an unambiguous gender presentation, so we restricted our pool to faces with a unanimous perception of being either male or female, according to subjective ratings norms from a U.S. rater sample [32,33]. Since young adults comprised our sample of interest, we excluded faces with a perceived age less than 18 or greater than 40. To reduce unintended differences in memory performance, we additionally excluded faces with an “unusualness” rating (from the normed data provided by [32], probing how likely they would stand out in a crowd) of more than two standard deviations above the normed mean.

Lastly, to increase diversity of the stimuli and avoid influences of race in trait or memory judgments, we used faces that were not unanimously one race. More specifically, we restricted the pool to faces in which the ratings for “AsianProb”, “BlackProb”, “LatinoProb”, “MultiProb”, “OtherProb”, and “WhiteProb” (columns found in the spreadsheet of subjective ratings norms, see [32,33]), were all less than or equal to 0.8, denoting at least some ambiguity in racial identity. Two final faces were removed from this set for being visual outliers that may be easier to remember given their dissimilarity to the other faces in the set (i.e., having blond/red hair). This resulted in a pool of 185 total faces (of 462 initially meeting the above criteria): 44 most perceived as Asian (27 males, 17 females), 32 most perceived as Black (18 males, 14 females), 80 most perceived as Latino (35 males, 45 females), and 29 most perceived as White (16 males, 13 females). Of these 185 faces, 96 were randomly drawn for each participant, constrained by the equal presentation of each perceived gender/race combination.

#### Sounds

The sounds used in this study came from the Free Open-Access Misophonia Stimuli database (FOAMS; [34]). These stimuli include multiple exemplars of assorted misophonia trigger sounds (i.e., not just oral/nasal), the categories of which were systematically chosen from a list of sounds most sensitive to misophonia severity (see [9,34]), making them good candidates for experimental study. Briefly, each trigger sound found in [9] to evoke discomfort that correlated significantly with misophonia severity was used as a search term on freesound.org, and the first ten searches to yield at least five instances that met pre-established criteria (e.g., at least four seconds of sound, no background noise) comprised the FOAMS initial release. These ten FOAMS categories include chewing gum, flipping newspaper, typing, dribbling a basketball, knife cutting food, human breathing, plastic crumpling, water drops, throat clearing, and swallowing. Additionally, an updated version of this stimulus set includes non-triggering controls sounds, all taken from freesound.org in the same manner. Across all sound categories, each exemplar was uploaded by a different user and intentionally varies in acoustic properties (e.g., duration, frequency, etc.). For more information on the sounds and the development of FOAMS, see [34] or zenodo.org/communities/miso-sound.

Although not all misophonic triggers are human-produced (e.g., [4,6,9]), for the present study we focused on human-produced sounds that could be presented concurrently with a face and reasonably imagined to be made by the person depicted. As such, we chose to use 8 of the available 10 trigger categories from FOAMS: 4 oral (hereafter “Oral”) categories (breathing, chewing gum, swallowing, throat clearing) and 4 non-oral (hereafter “Other”) categories (cutting food, dribbling a basketball, flipping newspaper, and typing). Additionally, to control for sound presentation more generally, we included 3 miscellaneous (hereafter “Control”) categories (coffee shop ambience, playing a harp, and using a hairdryer) as well as 1 category without sound (hereafter “Quiet”). Of note, we chose Control sounds that were similar to the Oral and Other categories in that they also involved human actions, but importantly they are sounds which are not commonly reported as misophonic triggers.

For each of the 11 sound categories (excluding Quiet), four different exemplars were chosen from the segmented audio files available through FOAMS. For instance, the “breathing” category included four unique audio clips of different breathing sounds. The segmentations were on average 4.75s in duration (range: 3.27–6.03s); segmentation lengths did not differ between sound categories (all *p*s > 0.05). The 44 segmented sounds were then normalized for amplitude using RMS mean in Adobe Audition CC (v14.4.0.38) for use in the present study.

### Procedure

The experiment was run in a dimly lit, sound-attenuated testing room using a Mac Mini computer with a 24-in. LCD monitor. Stimuli were presented using Python 3.8 and PsychoPy. Before beginning the experiment, participants were informed that they would be presented faces and asked to make judgments about the faces. Participants were made aware that sounds would play concurrently with the faces, and that some sounds may feel unpleasant to them. The experiment was broken down into two parts: Phase 1 (Learning) and Phase 2 (Memory).

All methods were approved by The Ohio State University Institutional Review Board, and all participants gave written informed consent to participate.

#### Phase 1: Learning

**Fig 2:**
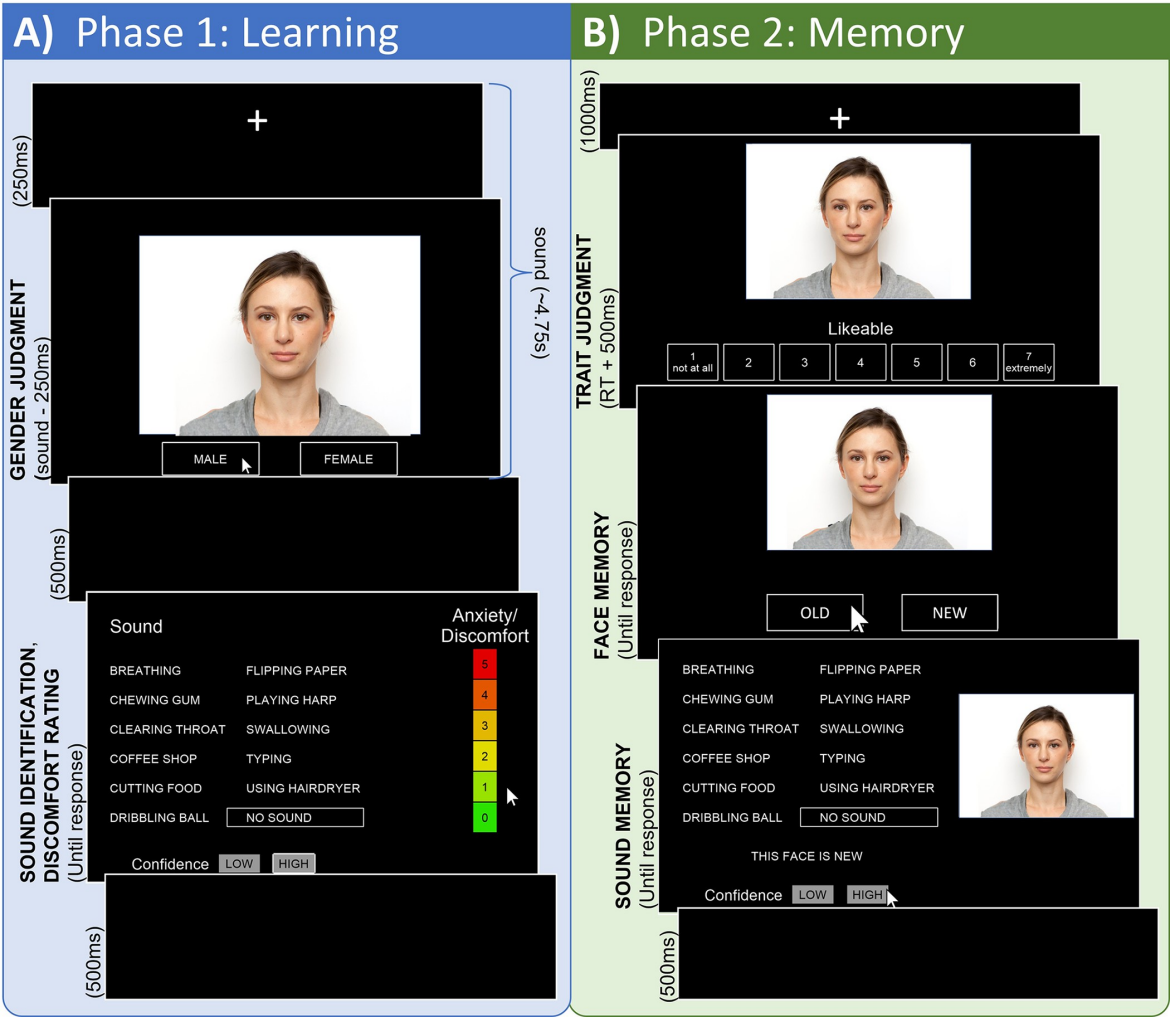
Method diagram. A) Phase 1: Sound begins during fixation. Face appears for the remainder of the sound length (~4.5s) with concurrent gender judgment. Participants click ratings of sound identity, confidence, and discomfort. B) Phase 2: Participants click likeable trait rating, old/new face memory judgment, and sound memory/confidence on separate screens. Note that the face shown is a mocked representation for depiction purposes only; experimental faces came from the Chicago Face Database (see Method: Stimuli).

In Phase 1 (Fig 2A), participants were shown 48 faces sequentially, each presented twice. Participants were instructed to judge the gender presentation of the face by clicking either “Male” or “Female”, as quickly and accurately as possible. During presentation of the face, a stimulus from one of the 12 sound categories played aloud through speakers. The face stayed on the screen throughout the duration of the sound, regardless of participant response. After the sound was finished (or after 4.75 seconds of no sound), participants were shown a response screen. They were given three additional tasks: 1) judge the identity of the sound they just heard, given one of the 12 available options (see Fig 2A); 2) assess their confidence in their identification of that sound between “Low” or “High”; and 3) rate their discomfort during the sound on a scale from 0 (no discomfort) to 5 (max discomfort). After clicking responses to all three questions, a “Continue” button appeared, after which participants started the next trial. Participants were given two practice trials (one male face, one female face) accompanied by a harp sound not used in the main experiment, then completed 96 experimental trials split into 4 blocks, between which they were offered short breaks.

The 48 faces learned in Phase 1 were equally split among gender and race (24 female, 24 male; 12 perceived as Asian, 12 as Black, 12 as Latino, and 12 as White). To improve memory performance, each face was introduced twice–once in the first half of the learning phase, and once in the second half. Each face was randomly assigned to one of the 12 sound categories for each participant and was presented with a different exemplar from that sound category on the two trials it was shown (e.g., Face #1 was presented with Breathing #1 in the first half of the trials and with Breathing #2 in the second half of the trials). This was done to mimic the incorporation of differential information about the face, as was done with multiple congruent behavioral statements in [21]. Additionally, this increased the possibility that at least one of the exemplars was recognizable as the target sound category and/or bothersome to misophonic participants, given the variability in participants’ experiences with triggering sounds. Similarly, to reduce effects of gender or race in memory judgments, each of the 48 sound stimuli were presented twice during Phase 1 –once with a female face and once with a male face, each from different perceived races. Phase 1 thus included 2 trials per sound exemplar (e.g., “breathing #1”), 8 trials per sound category (e.g., “breathing”), and 32 trials per condition (i.e., “Oral”).

#### Phase 2: Memory

In Phase 2 (Fig 2B), participants were given a surprise memory test of the faces and sounds to which they were just exposed. First, after a brief fixation, they were shown one of 96 faces (48 old, 48 new) sequentially. As with old faces, new faces were equally split among race and gender. Participants were asked to make 3 judgments about each face, on separate screens: 1) rate how likeable they found the person, on a scale from 1 (not at all likeable) to 7 (extremely likeable), similar to the rating scales used in previous trait assessments [21,32]; 2) judge whether they remembered seeing the face in Phase 1, by clicking either “Old” or “New”; and 3) recall what sound was initially playing if they encountered the face in Phase 1 given the same 12 options (see Fig 2B), as well as their confidence of that choice. The sound memory screen was shown regardless of participants’ old/new response, to reduce bias in choosing “New” to finish trials quicker; if the face was new and therefore did not have a learned sound, they were instructed to click “This face is new”. Participants completed 96 trials again split into 4 blocks for breaks. The 48 old trials were intermixed with new trials and included 4 trials per sound category and 16 per condition.

After completing Phase 2, participants were debriefed about misophonia and the goal of the experiment. They then completed demographic questions and the assessment scales mentioned above using Qualtrics survey software; the surveys were done last to avoid demand characteristics with sound ratings. The entire experiment took 40-70min to complete.

### Analyses

All data were de-identified prior to analysis. We used mixed ANOVAs, Student’s *t* tests, and Pearson’s correlations to assess differences in discomfort or performance between individuals with misophonia and controls. Effect sizes for *t* tests were calculated using Hedges’ *g* to correct for bias introduced by small samples (see [35]). Correlations involving misophonia level were conducted using S5 total scores, which contained a wider range and more individual variability than DVMSQ scores (e.g., fewer participants scoring 0, see Fig 1). For analyses in which multiple comparisons were conducted, *p*-values were corrected using the Holm-Bonferroni method [36] to control the familywise Type I error rate; corrected *p* values are denoted by *p*_HB_. All analyses were performed using Matlab (version R2021a) with addition of the functions dprime_simple.m [37] for face memory analyses (see below) and mes.m [38] for calculations of effect size.

#### Trial exclusions

To account for task-unrelated mind wandering or systematic response heterogeneity, trials with abnormally short or long response times (RTs) were removed for each participant. Specifically, any trial with a task response less than 100ms, greater than 30s (or 10s for gender judgment), or more than three standard deviations above the participant’s average RT for that task was removed. This resulted in an average of 4.81% of trials removed across participants (range: 1.56–12.5%); the amount of removed trials did not significantly differ between the misophonia and control groups (*t*(38) = -0.734, *p* = 0.467).

#### Discomfort

Since misophonic individuals vary in the categories and instances of sounds they find bothersome, we analyzed likeability ratings and memory performance both by our pre-determined sound categorization and by subjective report of sound discomfort by participants. For the latter, we took each participant’s discomfort ratings for all 96 trials in Phase 1 and calculated the upper and lower 10%. We classified sounds comprising the top 10% of discomfort ratings to be a participant’s “high discomfort sounds”, and sounds comprising the bottom 10% of discomfort ratings to be a participant’s “low discomfort sounds”. One participant rated all 96 sounds as 0 discomfort; in this case, all sounds were considered as “low discomfort sounds”. For a depiction of how frequently each sound stimulus ended up in a participant’s high vs. low discomfort category, see Supplement 2 (S1 Fig in S1 File).

#### Memory

For analyses of face memory, we calculated *d’*. In signal detection theory, *d’* provides a discrimination measure between a person’s ability to detect “signal” vs. “noise”; a value of 0 indicates an inability to discriminate signal from noise, whereas higher values indicate better ability to discriminate signal from noise (see [39] for a background on *d’* and signal detection theory). For this task, we are measuring participants’ abilities to report faces they had seen before (signal) as “Old” and identify distractor faces (noise) as “New”. Since *d’* cannot be calculated when responses are extreme (e.g., all faces reported as “Old”), calculations were adjusted using the loglinear approach to remove extreme values (i.e., hit rate or false alarm rate of 0 or 1) [39,40].

For analyses involving sound memory, we excluded participants who did not follow task instructions. Specifically, seven participants selected “This face is new” for fewer than 5% of the faces they reported as “New” in the face memory task, instead responding with “No Sound” for more than 95% of trials. (It is possible participants employed Phase 1 instructions and selected what sound they heard, since sound was never played in Phase 2.) This exclusion was needed to make calculations of signal detection unambiguous. Overall, *d’* values were overwhelmingly negative for sound memory across participants (signifying more false alarms than hits); we report hit rate in the results for interpretability, since the relationship between conditions matched the pattern observed with *d’*.

## Results

### Phase 1

#### Aim 1: Sound aversion (discomfort rating)

First, we sought to replicate prior work showing misophonic discomfort to non-oral trigger sounds. We did this by comparing discomfort ratings across sound categories and sounds individually, and then separated by sound identification accuracy. Lastly, we present subjective divisions of sounds reported as high vs. low discomfort.

**Fig 3:**
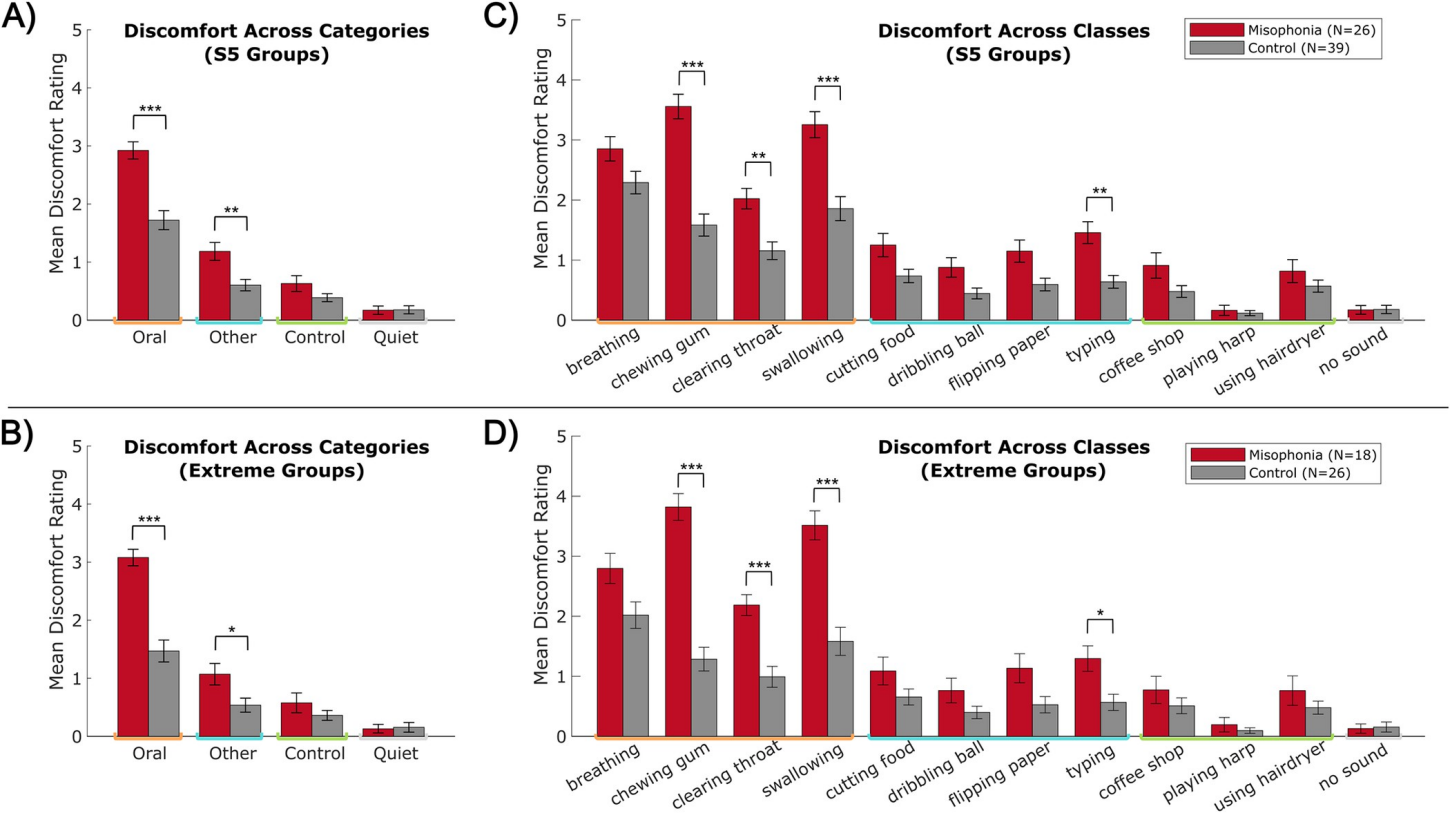
Discomfort rating differences between individuals with misophonia and controls. Left: across sound category, split by A) S5 groups and B) extreme groups. Right: across sound class, split by C) S5 groups and D) extreme groups. Orange = Oral sounds, blue = Other sounds, green = Control sounds, gray = Quiet. Error bars depict standard error of the mean. *p_HB_ ≤0.05, **p_HB_ ≤0.01, ***p_HB_ ≤0.001.

*Sound categories*. Using a 2 (group: misophonia vs. control, between-subjects) x 4 (sound category: Oral vs. Other vs. Control vs. Quiet, within-subjects) mixed ANOVA, we found significant main effects of group (*F*(1,252) = 35.228, *p* < 0.001, η_p_^2^ = 0.123), category (*F*(3,252) = 123.678, *p* < 0.001, η_p_^2^ = 0.596), and a group x category interaction (*F*(3,252) = 9.466, *p* < 0.001, η_p_^2^ = 0.101) (Fig 3A). Across all participants, there was a significant sequential decrease of discomfort across categories, from Oral to Other to Control to Quiet (Supplement 2, S2 Fig in S1 File left panel).

Crucially, while the same sequential decrease of discomfort was found within each group separately, there were differential ratings between groups depending on the sound category (Fig 3A). The misophonia group rated Oral sounds (*M* = 2.92, *SD* = 0.76;) and Other sounds (*M* = 1.18, *SD* = 0.79) significantly higher than controls did (Oral: *M* = 1.72, *SD* = 1.03; *t*(63) = 5.105, *p*_HB_ < 0.001, Hedges’ *g* = 1.277; Other: *M* = 0.60, *SD* = 0.61; *t*(63) = 3.339, *p*_HB_ = 0.004, Hedges’ *g* = 0.835), but there was no differences between groups in discomfort to Control sounds or Quiet. Analyses using extreme misophonia groups showed the same pattern of results (Fig 3B). Further, the discomfort ratings for Oral and Other sounds correlated significantly with S5 misophonia level across the entire sample (Oral: *r* = 0.58, *p*_HB_ < 0.001, Other: *r* = 0.41, *p*_HB_ = 0.002), but Control sound ratings and Quiet ratings did not vary with misophonia level (S2 Fig in S1 File right panels).

*Individual sound classes*. Additionally, given the variability in types of sounds reported as triggering to individuals with misophonia, we compared discomfort ratings between individual sound classes. Using a 2 (group: misophonia vs. control, between-subjects) x 12 (sound class: breathing vs. chewing vs. clearing throat vs. swallowing vs. cutting food vs. … vs. no sound, within-subjects) mixed ANOVA, we again found significant main effects of group (*F*(1,756) = 110.803, *p* < 0.001, η_p_^2^ = 0.128), class (*F*(11,756) = 69.887, *p* < 0.001, η_p_^2^ = 0.504), and a group x class interaction (*F*(11,756) = 6.819, *p* < 0.001, η_p_^2^ = 0.090) (Fig 3C). Probing the interaction further using pre-planned t-tests between groups for each class, we found particular classes whose discomfort significantly differed between the misophonia and control groups (Fig 3C): chewing gum (Misophonia: *M* = 3.56, *SD* = 1.04; Control: *M* = 1.58, *SD* = 1.15; *t*(63) = 7.048, *p*_HB_ < 0.001, Hedges’ *g* = 1.763), clearing throat (Misophonia: *M* = 2.02, *SD* = 0.87; Control: *M* = 1.16, *SD* = 0.92; *t*(63) = 3.810, *p*_HB_ = 0.003, Hedges’ *g* = 0.953), swallowing (Misophonia: *M* = 3.25, *SD* = 1.11; Control: *M* = 1.86, *SD* = 1.25; *t*(63) = 4.623, *p*_HB_ < 0.001, Hedges’ *g* = 1.157), and typing (Misophonia: *M* = 1.46, *SD* = 0.93; Control: *M* = 0.64, *SD* = 0.66; *t*(63) = 4.146, *p*_HB_ = 0.001, Hedges’ *g* = 1.037). The extreme groups likewise showed these differences (Fig 3D).

**Fig 4:**
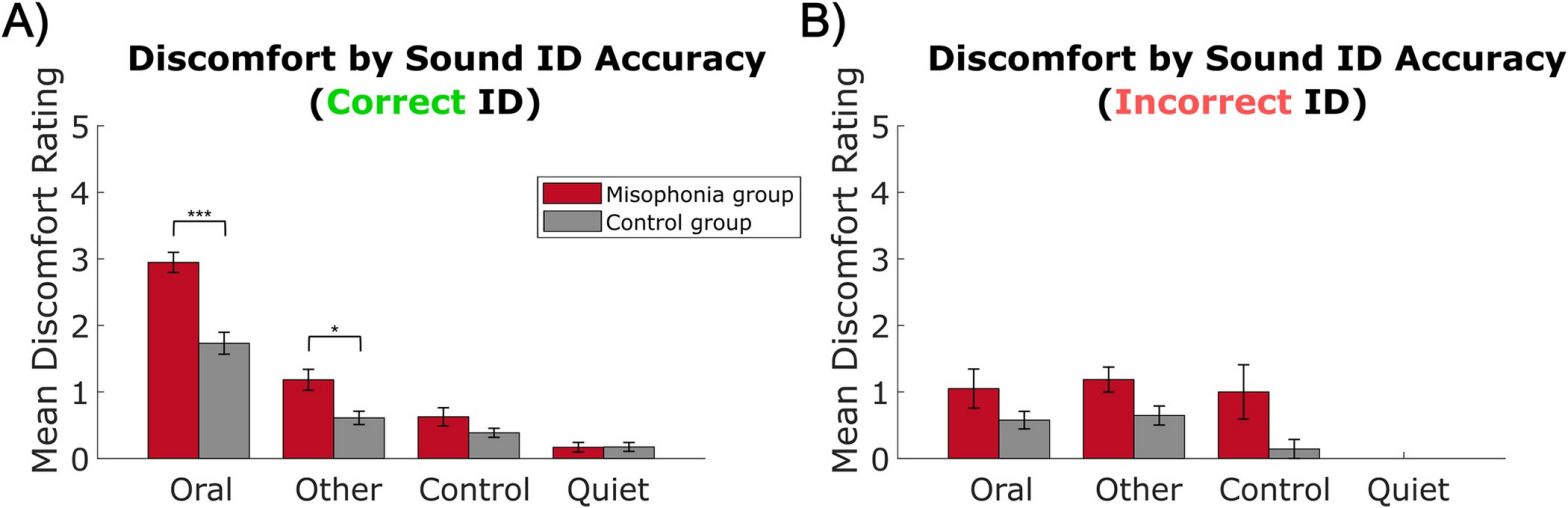
Discomfort ratings by sound ID accuracy, split by sound category. A) Discomfort for sounds correctly identified, B) discomfort for sounds incorrectly identified. Note that “Quiet” (i.e., no sound) was never incorrectly identified by any participant. Error bars depict standard error of the mean. *p_HB_ ≤0.05, ***p_HB_ ≤0.001.

*Sound identification*. Lastly, motivated by prior work demonstrating differential discomfort ratings based on sound identification, we analyzed reported discomfort by sound identification response. Note that participants varied in their sound identification accuracy within each category, so sample sizes vary between calculations; see Supplement 2 (S2 Table in S1 File) for a breakdown. Using a 2 (group: misophonia vs. control, between-subjects) x 4 sound category: Oral vs. Other vs. Control vs. Quiet, within-subjects) x 2 (sound identification: correct vs. incorrect) mixed ANOVA, we found significant interactions of group x category x identification (*F*(1,372) = 10.121, *p* = 0.002, η_p_^2^ = 0.026) and category x identification (*F*(3,372) = 13.658, *p* < 0.001, η_p_^2^ = 0.099) (Fig 4). To tease apart this effect, follow-up two-way ANOVAs were conducted within each sample separately, revealing significant category x identification interactions in both groups (misophonia: *F*(2,142) = 19.202, *p* < 0.001, η_p_^2^ = 0.213; control: *F*(2,221) = 13.479, *p* < 0.001, η_p_^2^ = 0.109). Both individuals with misophonia and controls reported Oral sounds as evoking more discomfort when the sound was correctly identified (Fig 4A, misophonia: *M* = 2.95, *SD* = 0.77; control: *M* = 1.73, *SD* = 1.03) compared to when the sound was incorrectly identified (Fig 4B, misophonia: *M* = 1.05, *SD* = 1.28; *t*(18) = 6.180, *p*_HB_ < 0.001, Hedges’ *g* = 1.832; control: *M* = 0.58, *SD* = 0.78; *t*(34) = 6.546, *p*_HB_ < 0.001, Hedges’ *g* = 1.243). Notably, when sounds were correctly identified (Fig 4A), the pattern of discomfort ratings matches that of Fig 3A and 3B, with misophonic individuals reporting significantly higher discomfort for Oral sounds (*M* = 2.95, *SD* = 0.77) and Other sounds (*M* = 1.18, *SD* = 0.80) compared to ratings by controls (Oral: *M* = 1.73, *SD* = 1.03; *t*(63) = 5.128, *p*_HB_ < 0.001, Hedges’ *g* = 1.283; Other: *M* = 0.61, *SD* = 0.62; *t*(63) = 3.233, *p*_HB_ = 0.014, Hedges’ *g* = 0.809), and no differences between groups in discomfort to Control sounds or Quiet. However, there were no significant differences between misophonic individuals and controls across categories when sounds were incorrectly identified (Fig 4B), suggesting misophonic discomfort to sounds is exacerbated by knowledge of what the sound is.

#### Aim 2: Task performance (gender judgment)

**Fig 5:**
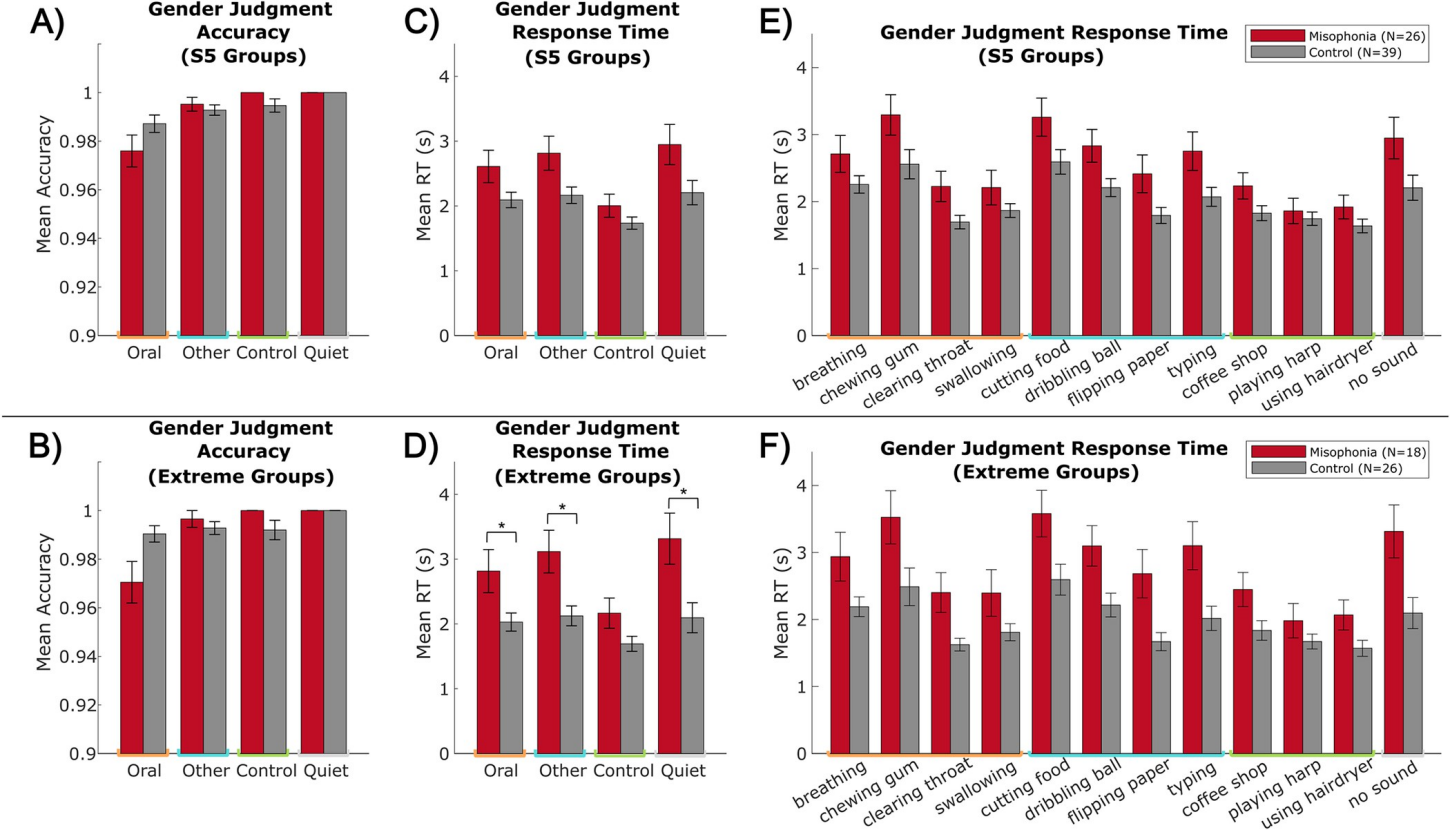
Gender judgment performance. Left: accuracy across sound categories, split by A) S5 groups and B) extreme groups. Middle: mean response time across sound categories, split by C) S5 groups and D) extreme groups. Right: mean response times across sound classes, split by E) S5 groups and F) extreme groups. Orange = Oral sounds, blue = Other sounds, green = Control sounds, gray = Quiet. Error bars depict standard error of the mean. Bars without error bars denote no variation in performance (e.g., all participants achieving 100% accuracy). *p_HB_ ≤0.05.

Next, we explored whether the presence of sounds alters real-time task performance by analyzing the accuracy and response times to the incidental encoding task. We calculated mean response times following the previously described trial exclusions. Using a 2 (group: misophonia vs. control, between-subjects) x 4 (sound category: Oral vs. Other vs. Control vs. Quiet, within-subjects) mixed ANOVA, a group x category interaction was assessed separately for both accuracy and response time (Fig 5).

While accuracy was at ceiling overall (mean = 99.13%), there was a significant main effect of sound category (*F*(3,252) = 14.916, *p* < 0.001, η_p_^2^ = 0.151) and a significant group x category interaction (*F*(3,252) = 2.942, *p* = 0.034, η_p_^2^ = 0.034) (Fig 5A). Accuracy on the gender judgment was lowest while performed during an Oral sound but did not significantly differ between individuals with misophonia and controls for any category, regardless of whether using S5 (Fig 5A) or extreme groups (Fig 5B).

Additionally, there were significant main effects of group (*F*(1,252) = 16.671, *p* < 0.001, η_p_^2^ = 0.062) and category (*F*(3,252) = 5.608, *p* < 0.001, η_p_^2^ = 0.063) in response time to the gender judgment (Fig 5C). Overall, participants were fastest during trials with Control sounds compared to the other three categories, and the misophonia group was significantly slower across all trials than the control group (*t*(63) = 2.197, *p*_HB_ = 0.032). Pre-planned independent samples *t*-tests revealed that individuals with misophonia were significantly slower than control participants at responding to the gender judgment during Oral sounds (Misophonia: *M* = 2.82s, *SD* = 1.41s; Control: *M* = 2.03s, *SD* = 0.71s; *t*(42) = 2.436, *p*_HB_ = 0.038, Hedges’ *g* = 0.733), Other sounds (Misophonia: *M* = 3.12s, *SD* = 1.40s; Control: *M* = 2.12s, *SD* = 0.78s; *t*(42) = 3.013, *p*_HB_ = 0.017, Hedges’ *g* = 0.907), and Quiet (Misophonia: *M* = 3.32s, *SD* = 1.68s; Control: *M* = 2.10s, *SD* = 1.18s; *t*(42) = 2.837, *p*_HB_ = 0.021, Hedges’ *g* = 0.854); however, these comparisons only reached significance in the extreme groups (Fig 5D).

As with discomfort ratings, we further compared response times between individual sound classes. Using a 2 (group: misophonia vs. control, between-subjects) x 12 (sound class: breathing vs. chewing vs. clearing throat vs. swallowing vs. cutting food vs. … vs. no sound, within-subjects) mixed ANOVA, we again found significant main effects of group (*F*(1,756) = 43.690, *p* < 0.001, η_p_^2^ = 0.055) and class (*F*(11,756) = 8.758, *p* < 0.001, η_p_^2^ = 0.113) (Fig 5E). However, there were no significant between-group differences within any individual sound class after correcting for multiple comparisons, in either the S5 (Fig 5E) or extreme groups (Fig 5F).

Additional analyses on data from the gender judgment task can be found in Supplement 3. We calculated median RT post hoc (S3 Fig in S1 File) and found a similar pattern of results as presented in Fig 5. Of note, whereas using mean RTs did not reveal significant between-group differences within any individual sound class, results using median RTs did reach significance for a subset of classes (see S3 Fig in S1 File bottom right panel). We further depict accuracy data collapsed across groups (S4 Fig in S1 File left panel), and show that misophonia level correlates significantly with mean RT during Other sounds (*r* = 0.32, *p*_HB_ = 0.033) and marginally for the other three categories (S4 Fig in S1 File right panels).

### Phase 2

#### Aim 3: Trait judgment (likeability rating)

In the memory phase, we first explored whether trigger sounds influenced subjective trait judgments of the face stimuli. We did this by comparing the likeability ratings assigned to the learned faces, both between our predetermined sound categories and between each participant’s high vs. low discomfort sounds. Lastly, to determine if source memory for the face influences trait judgments, we present likeability ratings split by accuracy of reporting the sound paired with the face.

*Sound categories*. A 2 (group: misophonia vs. control, between-subjects) x 5 (sound category: New Faces vs. Oral vs. Other vs. Control vs. Quiet, within-subjects) mixed ANOVA revealed no significant main effects or interactions; likeability ratings did not significantly vary by misophonia level for any category (S5 in S1 File). No differences were found for raw ratings or z-scored ratings, so we depict raw ratings for easier interpretation.

**Fig 6:**
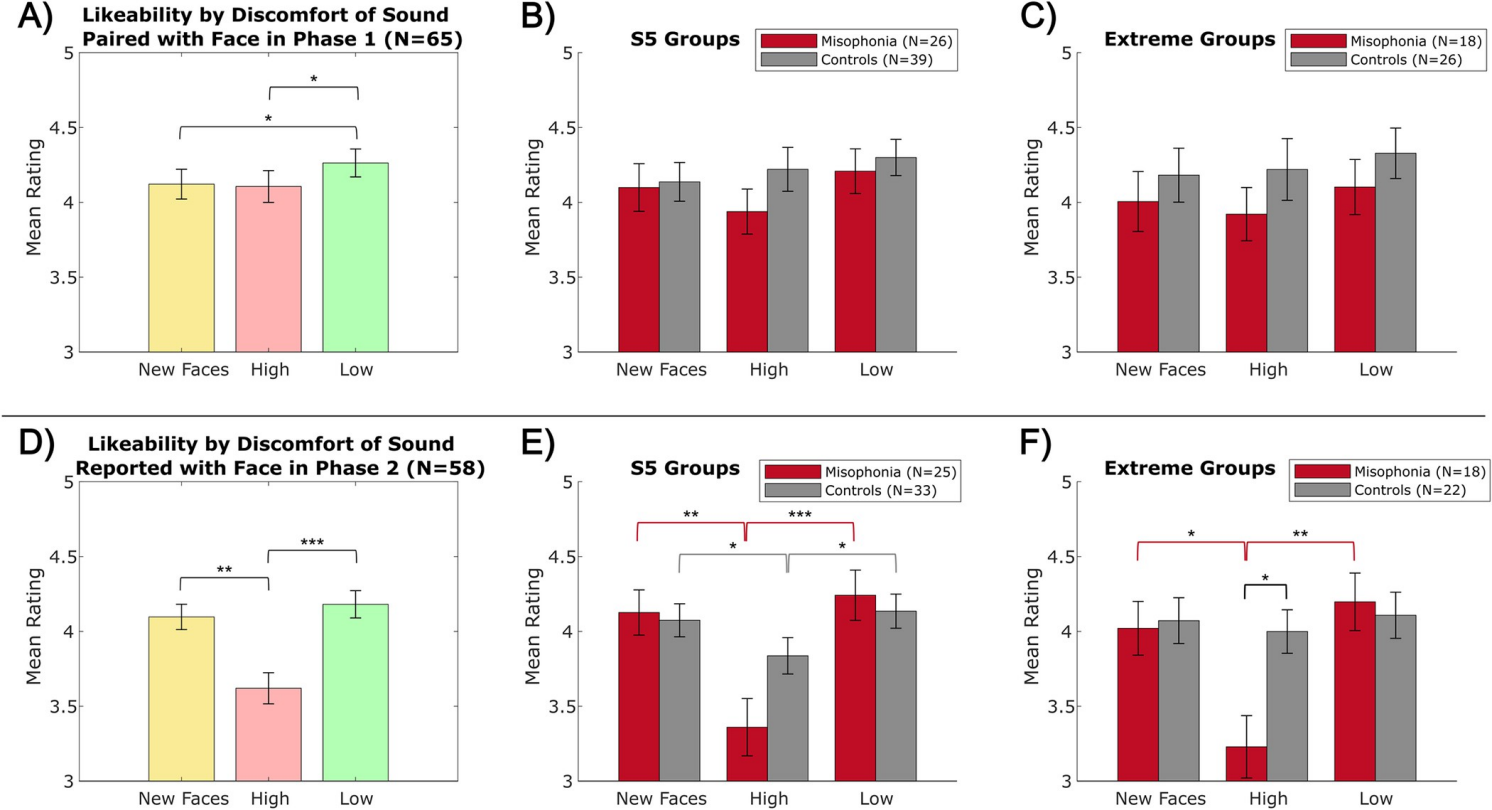
Likeability ratings by subjective sound discomfort. Top: Ratings for faces originally paired with sounds reported as high/low discomfort in Phase 1, A) collapsed across all participants, B) split by S5 groups, C) split by extreme groups. Bottom: Ratings for faces in which a high/low discomfort sound was reported from memory in Phase 2, D) collapsed across all participants, E) split by S5 groups, F) split by extreme groups. Error bars depict standard error of the mean. †p_HB_≤0.10, *p_HB_ ≤0.05, **p_HB_ ≤0.01, ***p_HB_ ≤0.001.

*Sound discomfort*. Probing further, we analyzed trait judgments based on self-reported discomfort. As a reminder, one participant self-reported 0 discomfort for all sounds (see Analyses) and thus is not included in comparisons with high discomfort sounds. Across all participants, faces paired with sounds reported as low discomfort (*M* = 4.26, *SD* = 0.75) were rated as significantly more likeable than both new faces (*M* = 4.12, *SD* = 0.80; *t*(64) = -2.615, *p*_HB_ = 0.033, Hedges’ *g* = -0.181) and faces paired with high discomfort sounds (*M* = 4.10, *SD* = 0.86; *t*(63) = -2.618, *p*_HB_ = 0.033, Hedges’ *g* = -0.194) (Fig 6A). Trait judgments based on self-reported discomfort did not significantly differ between groups using either S5 (Fig 6B) or extreme (Fig 6C) divisions.

Additionally, we analyzed the influence of sound memory on likeability ratings, regardless of accuracy. As a reminder, seven participants did not follow (or misinterpreted) instructions for the sound memory question (see Analyses) and thus were not included in these comparisons. Moreover, two further participants never reported a sound they rated as high discomfort in Phase 1 as their sound memory response in Phase 2, and therefore are not included in comparisons with high discomfort sounds. Overall, when participants reported a high discomfort sound (*M* = 3.62, *SD* = 0.84) as having been paired with the face, they rated that face as significantly less likeable than either faces with low discomfort memories (*M* = 4.18, *SD* = 0.74; *t*(54) = -5.864, *p*_HB_ < 0.001, Hedges’ *g* = -0.708) or new faces (*M* = 4.10, *SD* = 0.68; *t*(54) = 4.985, *p*_HB_ < 0.001, Hedges’ *g* = 0.621) (Fig 6D). Notably, this effect was driven by the misophonia group (see Fig 6E and 6F), most clearly demonstrated by the extreme groupings (Fig 6F): individuals with misophonia reported significantly lower likeability ratings for high discomfort memories (*M* = 3.23, *SD* = 0.88) compared to low discomfort memories (*M* = 4.20, *SD* = 0.82; *t*(17) = -4.549, *p*_HB_ = 0.002, Hedges’ *g* = -1.113) and new faces (*M* = 4.02, *SD* = 0.76; *t*(17) = 3.432, *p*_HB_ = 0.016, Hedges’ *g* = 0.938), and gave significantly lower likeability ratings for high discomfort memories than the control group did (*M* = 4.00, *SD* = 0.67; *t*(37) = -3.097, *p*_HB_ = 0.011, Hedges’ *g* = -0.974).

*Sound memory*. Lastly, we looked at the influence of sound memory accuracy on likeability ratings using a 2 (group: misophonia vs. control, between-subjects) x 4 (sound category: Oral vs. Other vs. Control vs. Quiet, within-subjects) x 2 (sound memory accuracy: correct vs. incorrect, within-subjects) mixed ANOVA. Note that participants varied in their sound memory accuracy within each category, so sample sizes vary between calculations; see Supplement 4 (S3 Table in S1 File) for a breakdown. Regardless, we found a significant main effect of category (*F*(3,386) = 4.482, *p* = 0.004, η_p_^2^ = 0.034) and a significant category x accuracy interaction (*F*(3,386) = 18.323, *p* < 0.001, η_p_^2^ = 0.047).

**Fig 7:**
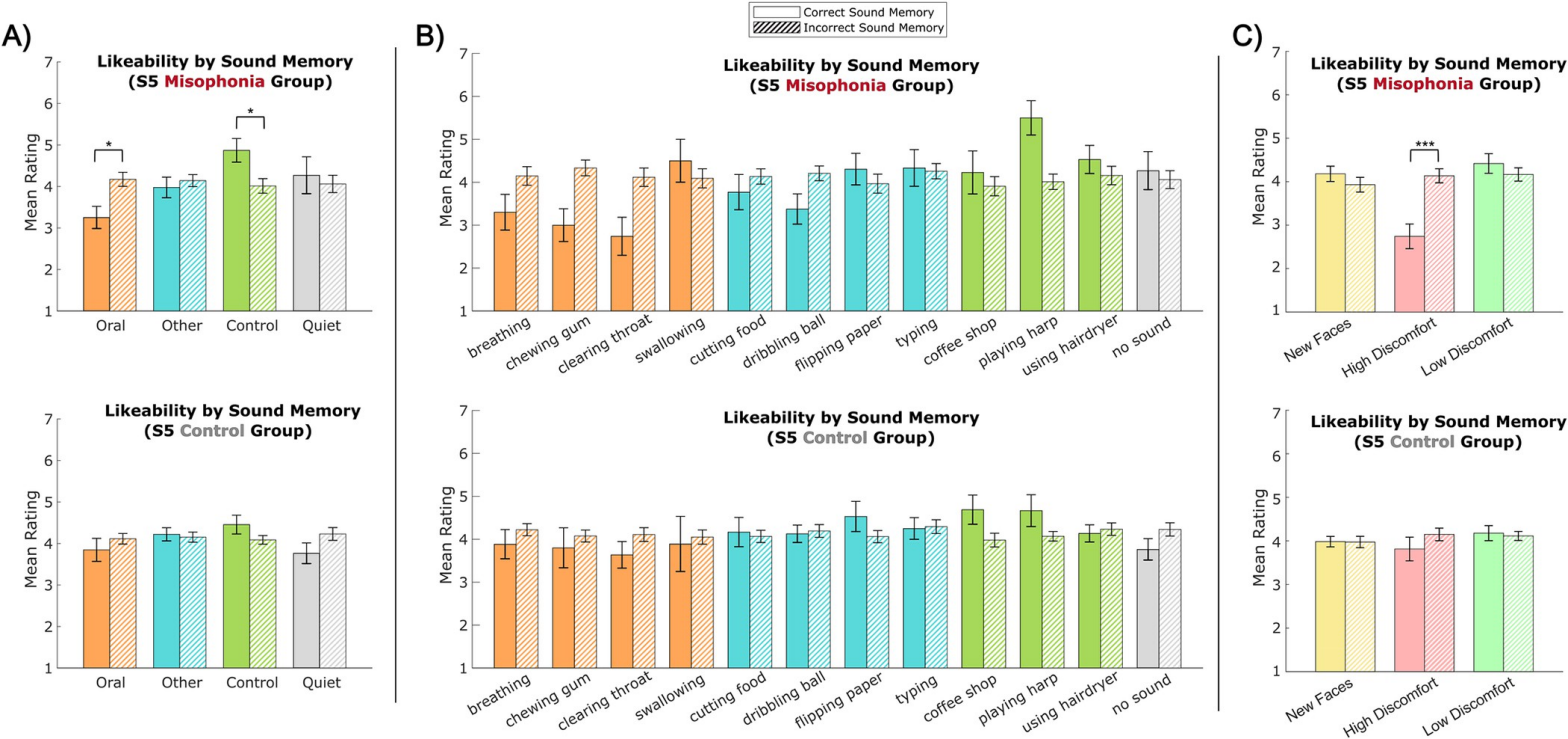
Likeability ratings by sound memory performance. A) across sound categories, B) across sound classes, C) across sound discomfort. (top) S5 misophonia group, (bottom) S5 control group. Solid bars denote ratings when the sound was correctly remembered, and hashed bars denote ratings when the sound was incorrectly remembered. Error bars depict standard error of the mean. *p_HB_ ≤0.05, ***p_HB_ ≤0.001.

To tease apart this effect, follow-up two-way ANOVAs were conducted within each sample separately (Fig 7A), revealing no significant main effects or interactions in the control group but a significant main effect of category (*F*(3,169) = 3.458, *p* = 0.018, η_p_^2^ = 0.058) and a significant category x accuracy interaction (*F*(3,169) = 6.070, *p* = 0.002, η_p_^2^ = 0.085) in the misophonia group. Individuals with misophonia gave significantly lower likeability ratings for faces paired with Oral sounds when they correctly remembered the sound (*M* = 3.25, *SD* = 1.19) compared to when they incorrectly remembered the sound (*M* = 4.17, *SD* = 0.84; *t*(19) = -2.894, *p*_HB_ = 0.037, Hedges’ *g* = -0.889). In contrast, individuals with misophonia gave significantly higher likeability ratings for faces paired with Control sounds when they correctly remembered the sound (*M* = 4.87, *SD* = 1.27) compared to when they incorrectly remembered the sound (*M* = 4.01, *SD* = 0.88; *t*(19) = 2.862, *p*_HB_ = 0.037, Hedges’ *g* = 0.789).

When analyzed across sound classes (Fig 7B), the same significant ANOVA main effects/interactions were observed; see S4 Table in S1 File for calculation sample sizes. While likeability ratings numerically varied (e.g., lower likeability for chewing gum and clearing throat, higher likeability for playing harp), none of the individual classes reached significance when correcting for multiple comparisons.

When analyzed across sound discomfort (Fig 7C), the same significant ANOVA main effects/interactions were again observed; see S5 Table in S1 File for calculation sample sizes. Individuals with misophonia gave significantly lower likeability ratings for faces paired with sounds they rated as high discomfort when they correctly remembered the sound (*M* = 3.25, *SD* = 1.19) compared to when they incorrectly remembered the sound (*M* = 4.17, *SD* = 0.84; *t*(19) = -2.894, *p*_HB_ = 0.037, Hedges’ *g* = -0.889). Faces paired with high discomfort sounds that were incorrectly remembered did not differ in reported likeability to faces paired with incorrectly remembered low discomfort sounds or incorrectly remembered new faces. No significant differences from sound memory accuracy were observed in the control group.

#### Aim 4: Memory performance

Finally, we investigated whether sounds presented during learning affected subsequent memory performance. We did this by comparing face memory (adjusted *d’*) between each sound category and between high vs. low discomfort sounds. We then explored source memory by analyzing memory for the sound originally paired with each learned face.

*Face memory*: *Sound categories*. A 2 (group: misophonia vs. control, between-subjects) x 4 (sound category: Oral vs. Other vs. Control vs. Quiet, within-subjects) mixed ANOVA revealed only a significant main effect of group (*F*(1,252) = 9.268, *p* = 0.003, η_p_^2^ = 0.035), with misophonic individuals performing better than control individuals. While memory performance was worse for Oral sounds across participants, face memory did not significantly vary by misophonia level for any category (Supplement 5, S7 Fig in S1 File).

**Fig 8:**
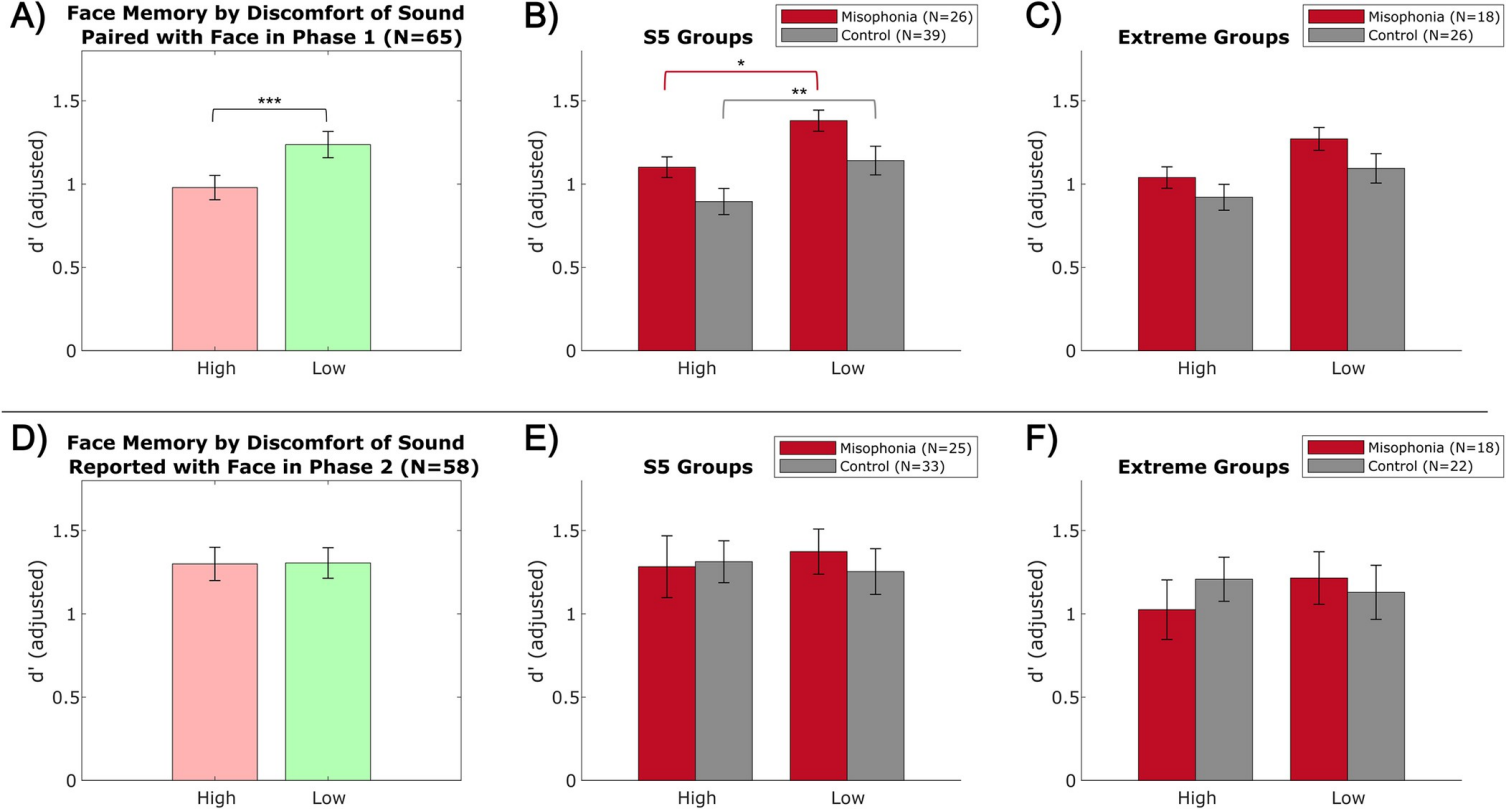
Face memory performance by subjective sound discomfort. Top: Memory for faces originally paired with sounds reported as high/low discomfort in Phase 1, A) collapsed across all participants, B) split by S5 groups, C) split by extreme groups. Bottom: Memory for faces in which a high/low discomfort sound was reported from memory in Phase 2, D) collapsed across all participants, E) split by S5 groups, F) split by extreme groups. Error bars depict standard error of the mean. *p_HB_ ≤0.05, **p_HB_ ≤0.01, ***p_HB_ ≤0.001.

*Face memory*: *Sound discomfort*. Probing further, we again analyzed face memory based on subjective report of sound discomfort by participants, rather than our pre-determined categorization (Fig 8). Across all participants, faces paired with sounds reported as high discomfort (*M* = 0.98, *SD* = 0.59) were remembered significantly less than faces paired with sounds reported as low discomfort (*M* = 1.24, *SD* = 0.63; *t*(63) = -5.066, *p*_HB_ < 0.001, Hedges’ *g* = -0.420) (Fig 8A). This pattern held true in both groups separately when split using the S5 (Fig 8B), but not in the extreme groups (Fig 8C). There was no overall difference in face memory based on the discomfort of the sound reported with the face, ignoring accuracy of the sound report (Fig 8D–8F).

**Fig 9:**
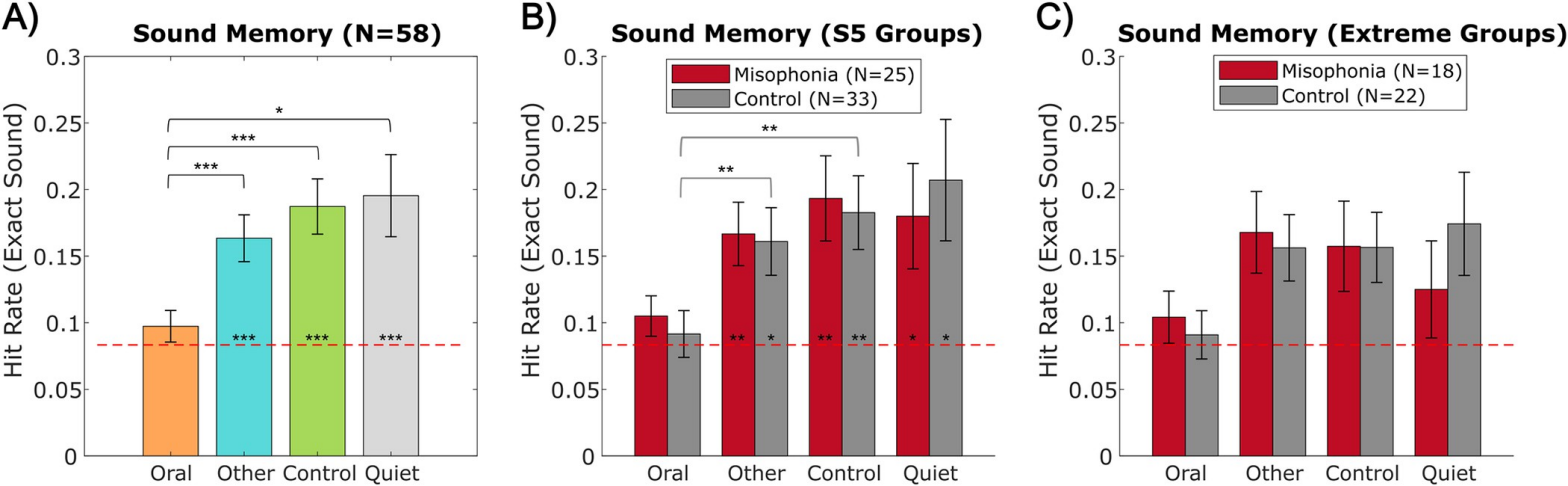
Sound memory performance by sound category. A) collapsed across all participants, B) split by S5 groups, C) split by extreme groups. Red dotted line (y = 0.083) represents chance performance, with asterisks inside bars denoting performance relative to chance. Error bars depict standard error of the mean. *p_HB_ ≤0.05, **p_HB_ ≤0.01, ***p_HB_ ≤0.001.

*Sound memory*. Across the 48 “Old” trials, overall mean sound memory accuracy was 0.15 (*SD* = 0.20). Given 12 sound options on the selection screen to choose from, selecting the correct sound by chance for a face remembered as “Old” is 0.083 (1/12). Although a difficult task, participants performed better than chance at remembering Other sounds (*M* = 0.16, *SD* = 0.13; *t*(57) = 4.559, *p*_HB_ < 0.001, Hedges’ *g* = 0.936), Control sounds (*M* = 0.19, *SD* = 0.16; *t*(57) = 5.011, *p*_HB_ < 0.001, Hedges’ *g* = 1.011), and Quiet (*M* = 0.20, *SD* = 0.23; *t*(57) = 3.633, *p*_HB_ = 0.001, Hedges’ *g* = 0.730); however, participants were at chance for identifying Oral sounds (*M* = 0.10, *SD* = 0.09; *t*(57) = 1.179, *p*_HB_ = 0.243) (Fig 9A). This effect was found in both groups separately when split using the S5 (Fig 9B), but not in the extreme groups (Fig 9C).

A 2 (group: misophonia vs. control, between-subjects) x 4 (sound category: Oral vs. Other vs. Control vs. Quiet, within-subjects) mixed ANOVA revealed a main effect of category (*F*(3,224) = 4.030, *p* = 0.008, η_p_^2^ = 0.051) (Fig 9B). Specifically, Oral sounds were remembered significantly worse than all other categories (Other: *t*(57) = -4.406, *p*_HB_ < 0.001, Hedges’ *g* = -0.575; Control: *t*(57) = -4.544, *p*_HB_ < 0.001, Hedges’ *g* = -0.694; Quiet: *t*(57) = -2.946, *p*_HB_ = 0.019, Hedges’ *g* = -0.547), but the other categories were not significantly different from each other. There were no significant differences in sound memory between individuals with misophonia and controls in either the S5 groups (Fig 9B) or extreme groups (Fig 9C), and analyzing memory errors did not reveal a notable pattern.

## Discussion

In this experiment, we asked: when exposed to triggering sounds, does misophonia impact discomfort rating, task performance, likeability of the trigger-associated person, or memory for the situation? Our results corroborate previous misophonia research in a few ways. First, as demonstrated by [9], misophonic aversion extends past discomfort to oral/nasal sounds. Indeed, using completely new sound stimuli and samples of participants, we replicate the differential ratings of discomfort between individuals with misophonia and controls for both oral and non-oral sounds. Looking at sound classes individually adds nuance to this finding: while group differences in ratings to Other sounds seem to be mainly driven by significant differences in response to typing, we note that all three of the Other sound classes likewise were accompanied by between-group differences (*p*s < 0.05) before correcting for multiple comparisons. Interestingly, this was not the case for breathing (*p* > 0.05). This adds additional evidence that 1) more than just oral/nasal sounds are bothersome in misophonia (and not all oral/nasal sounds are uniquely aversive to individuals with misophonia), and 2) misophonia is not a general sound aversion or hearing disorder, since control sounds were not differentially rated.

Further, asking participants to identify each sound enabled us to study the effect of sound identification on experienced discomfort. [41] found that participants with misophonia were most bothered by sounds once they could identify them from masking noise. [16,20] showed that an individual’s perception of a sound’s source (i.e., triggering vs. non-triggering) influenced how aversive they found the sound to be. Similarly, [9] found that participants with misophonia rated oral sounds more aversive when they were correctly identified, but non-oral/nature sounds less (or not) aversive when correctly identified. In the present experiment, we found similar results: the misophonia group rated correctly identified Oral sounds with higher discomfort than incorrectly identified Oral sounds, but did not significantly differ in discomfort ratings across identification of Other or Control sounds. Since fewer participants actually reported an Other or Control sound incorrectly, we expect we did not have enough power to detect effects for these analyses (Other sounds: observed power = 0.17; Control sounds: observed power = 0.35). Interestingly, this pattern of higher discomfort for correctly identified Oral sounds vs. incorrectly identified Oral sounds also held true for control participants, perhaps suggesting that sound identity modulates discomfort across the general population and is merely exacerbated in misophonia. Moreover, when sounds were incorrectly identified, individuals with misophonia no longer significantly differ from controls in their discomfort ratings for Oral and Other sounds. This finding further supports the idea that misophonic aversion is not solely driven by the auditory stimulus itself; if it were, we would have seen a similar pattern of discomfort ratings regardless of sound identification. These data add more credence to the phenomenon that knowledge of the sound’s identity impacts misophonic aversion.

Additionally, our data show a general performance deficit for misophonic individuals on tasks as basic as gender judgment. While accuracy was more or less at ceiling across trials and did not significantly differ between misophonic individuals and controls, significantly slower response times were found for misophonic individuals across both Oral and Other categories, supporting our claim that more than just oral sounds impact misophonia. The difference in response time on trials with no sound was not expected, but might be explained by an anticipatory pause by individuals with misophonia as they waited for a potentially triggering sound to play; it is possible that only once they were confident no sound was happening did they shift their attention to the gender judgment task. Comparable to conclusions of [14], the cognitive effort required to split attention between the sound and the face and prioritize face information may have been more resource-demanding to individuals with misophonia. This provides additional evidence for daily challenges in multi-tasking or completion of tasks when in the presence of certain stimuli, and highlights that task impairments are not exclusive to Oral sounds.

In contrast to our predictions based on previous literature, however, we found no evidence of differential trait ratings to Oral or Other sounds in misophonia. Focusing on individualized divisions of triggers vs. non-triggers (instead of our predetermined sound categories) revealed more nuanced patterns of results. Specifically, when a face was paired with a sound that a participant rated as evoking low discomfort, they rated that face as more likeable than a new face they hadn’t seen before. This may be evidence of the mere exposure effect, a phenomenon in which liking for a stimulus increases following repeated exposures of that stimulus [42,43]; at the time of the trait rating, participants were seeing old faces for the third time. However, this phenomenon does not fully explain the results, as faces paired with high discomfort sounds were not rated as more likeable than new faces, nor were old faces rated as more likeable than new faces in general. Additional analyses of discomfort revealed that participants may have internally used sound pairings as a strategy for assessing likeability, or as a rationale for their likeability ratings. Specifically, when misophonic participants reported a sound that they rated as high discomfort as their sound memory answer in Phase 2, they found that face to be significantly less likeable than when they reported a low discomfort sound, and to a greater extent than controls. Interestingly, this effect shifts with a more liberal classification of high vs. low discomfort (using a 25% cutoff instead of a 10% cutoff), whereby controls no longer show a difference in likeability ratings between high and low discomfort sound memories (S6 Fig in S1 File bottom panels). This tells us that when individuals view a face they don’t like, they’re internally attributing the dislike to the face’s association with a bothersome sound; misophonic individuals just have a lower threshold for sound aversion. It also demonstrates an added utility of including multiple sound exemplars in misophonia experiments and personalizing trigger analyses for each individual, since no effect was seen in likeability ratings when using our external sound categories.

In addition to differences in likeability ratings depending on sound discomfort, we also found differences in likeability ratings depending on sound memory. When individuals with misophonia correctly remembered that an Oral sound was paired with a face, they found that face to be less likeable, whereas when they correctly remembered that a Control sound (e.g., harp) was paired with the face, they found that face to be more likeable. The same effect was observed dividing sounds by subjective discomfort: individuals with misophonia found faces paired with sounds they rated as high discomfort to be less likeable, but only when they correctly remembered the high discomfort sound. In contrast, a lack of source memory for the face (i.e., incorrect sound response) was associated with an average and non-significantly different likeability rating across categories and discomfort levels. This finding suggests that trait judgments are impacted by explicit source memory in misophonia, but perhaps not by implicit memory or prior experience with a person.

Lastly, while we found overall differences in memory depending on the sound category originally introduced with the face, with faces paired with Oral sounds remembered worse than faces paired with Control sounds or Quiet, this effect was not affected by misophonia. More generally, faces paired with high discomfort sounds were remembered worse than faces paired with low discomfort sounds, but this effect again did not differ between the misophonia and control groups. In general, faces learned during Quiet were remembered the best, in line with prior work showing auditory distraction in general affects memory performance [24]. This pattern was also present in sound memory: regardless of misophonia, participants had a lower hit rate at reporting Oral sounds compared to all other categories, and a lower hit rate at high discomfort sounds versus low discomfort sounds.

Why were there no trait judgment or memory differences to the sound categories that were specific to misophonia? We see three possible explanations. First, perhaps individuals with misophonia have a subjective feeling of being impaired by their sound sensitivity, but in actuality are performing (through compensatory mechanisms, for instance) equivalent to their non-misophonic peers. This explanation seems less likely, given prior work showing memory deficits due to sound sensitivity (e.g., [10]).

Second, perhaps different subsets of misophonia exist, such that trigger exposure affects cognitive performance in each subset differently and thus wash out when analyzed together. For instance, some individuals may be impaired by trigger sounds, leading to distraction during current task goals and worse memory for the situation later on. Others, however, may see memory improvement for situations involving trigger sounds, due to a heightened emotional experience of the situation and thus an emotional enhancement of memory (see [44]). Indeed, our data showed large variation in individual *d’*s for Oral and/or high discomfort sounds in particular, with some individuals (5 with misophonia, 16 controls) boasting higher memory for faces paired with Oral/discomforting sounds than with Control/Quiet, and other individuals (21 with misophonia, 22 controls) clocking worse memory for Oral/discomforting sounds than for Control/Quiet. However, none of the demographic or clinical measures we collected (e.g., age, gender, OCD level, depression, stress, anxiety, or individual misophonia assessment subscales) explained the variation in memory performance, and the resultant group sizes within each misophonia x memory subdivision (specifically individuals with misophonia with better memory for faces paired with high discomfort sounds) were too small to make a meaningful statistical comparison. Thus, the possibility of misophonic subsets needs to be explored in future research.

Third, and perhaps not mutually exclusive to the other explanations, perhaps the current task design was not powerful enough to detect trait judgment or memory differences in misophonia. For instance, perhaps if we had asked participants to rate likeability for each face prior to sound exposure, we would have obtained a more accurate assessment of how sound exposure altered that rating. Similarly, while the 1–7 response screen was modelled off prior work with this dataset and others (e.g., [21,32]) and our instructions encouraged participants to use the full scale, our participants largely stuck to average ratings for each face. Thus, it is possible that a more sensitive response method or an alternative trait probe could better clarify if differences in social perception exist. Further, perhaps explicit participant instruction to memorize the faces would have better elucidated group differences in long-term memory. Many participants anecdotally shared that they were caught off guard by the memory phase and felt they performed poorly; it is possible that adjusting a participant’s goals during face learning from “identify the face gender and sound” to “memorize the face” would better demonstrate just how impacted by misophonia memory performance is. In sum, future memory experiments with misophonic individuals might consider: 1) a larger sample size, for a more equal distribution of better vs. worse memory performance; 2) an explicit memory instruction; and/or 3) a pre- and post-test of memory to quantify changes following trigger exposure more accurately.

Regardless, this experiment has several limitations that must be kept in mind. First, we did not have a clinician or audiologist specifically assess misophonia or other related auditory disorders (e.g., hyperacusis, tinnitus) in our participants. We observed notable inconsistencies in how individuals responded to the two misophonia assessment surveys or the solo question “Do you think you have misophonia?”. Until a single misophonia questionnaire is widely accepted by the field, we urge researchers and clinicians to incorporate multiple forms of assessment in their work to get a more complete picture of each participant’s experiences and not bias results by defining groups in a particular way. We also must consider the ramifications of a participant self-reporting “No” to having misophonia, and thus being excluded from further survey questions that rely on an affirmative initial response, but aligning with misophonic symptoms when forced to complete questionnaires in full. To navigate these inconsistencies, we employed a conservative metric post hoc to define extreme groups (i.e., congruency in all three metrics) but this resulted in the misophonia and control samples being smaller and demographically less matched than we initially intended. The misophonia group ended up primarily white and female-identifying, whereas the control group was primarily male-identifying. While prior work is split on whether misophonia is more prevalent in the female sex [25] or not differentially affected by sex [41,45], multiple studies have found higher rates of misophonia in female-identifying participants (e.g., [8,25,46]). Nevertheless, the present manuscript is not well-suited to characterize differences in social/cognitive performance or prevalence of misophonia as a function of demographic factors; future work specifically examining these differences in misophonia would be beneficial.

Additionally, the experiment is limited in the categories of stimuli used. The sounds in the Control category (i.e., coffee shop, playing harp, using hairdryer) varied widely in acoustic and emotional content and were not explicitly matched to the experimental sounds (as was done in [16]). As such, we cannot completely rule out low-level acoustic effects. Similarly, the experimental sounds did not cover the wide range of triggers that our misophonic sample endorsed via surveys. For example, many of our participants self-reported being most bothered by sounds like slurping or sniffling, which were not represented in the present study. To maximize the chance of observing an effect, future work should seek to either A) expand the number of included stimulus categories to ensure some trigger overlap with each participant, or B) personalize the stimulus categories for each participant so only their most/least triggering sounds are included in the experiment. Regardless, we feel the methods and results presented here add to our understanding of misophonic impairment and invite more study into the cognitive and social ramifications of misophonia.

## Supporting information

S1 File Supplementary information. Contains all Supplementary tables and figures referenced in the manuscript.
(DOCX)
