# Supplementary material for: The effect of misophonia on cognitive and social judgments
Source: PLoS One. 2024 May 9;19(5):e0299698. doi: 10.1371/journal.pone.0299698 (PMC11081244; doi:10.1371/journal.pone.0299698)
Supplement: S1 File — Contains all Supplementary tables and figures referenced in the manuscript. (DOCX) [file pone.0299698.s001.docx]

# Supplementary Material

## Supplement 1: Misophonia Assessments

**Supplementary Table S1.** Demographics and questionnaire scores for misophonia and control groups.

|  |  |  |  |  | **DVMSQ** | | | | | **S5** | | | | | |
| --- | --- | --- | --- | --- | --- | --- | --- | --- | --- | --- | --- | --- | --- | --- | --- |
| **Group** | **Age** | **Gender** | **Race** | **Self- report** | **Symp** | **Impair** | **TOTAL** | **Diag** | **Diag (adj)** | **Ext** | **Int** | **Imp** | **Out** | **Thr** | **TOTAL** |
| Miso* | 19 | NB | B | Yes | 35 | 13 | 48 | Clin | Clin | 22 | 34 | 25 | 14 | 39 | 134 |
| Miso* | 25 | F | W | Yes | 32 | 17 | 49 | Clin | Clin | 25 | 42 | 24 | 27 | 47 | 165 |
| Miso* | 20 | NB | W | Yes | 35 | 23 | 58 | Clin | Clin | 4 | 33 | 31 | 19 | 42 | 129 |
| Miso* | 35 | F | W | Yes | 33 | 9 | 42 | Clin | Clin | 41 | 40 | 39 | 12 | 46 | 178 |
| Miso* | 19 | F | W | Yes | 34 | 14 | 48 | Clin | Clin | 36 | 37 | 26 | 34 | 37 | 170 |
| Miso* | 19 | F | W | Yes | 29 | 5 | 34 | Clin | Clin | 46 | 48 | 9 | 10 | 49 | 162 |
| Miso* | 25 | F | A | Yes | 26 | 12 | 38 | None | Clin | 27 | 20 | 11 | 2 | 32 | 92 |
| Miso* | 30 | NB | W | Yes | 34 | 9 | 43 | Clin | Clin | 35 | 1 | 8 | 10 | 44 | 98 |
| Miso* | 19 | F | W | Yes | 30 | 16 | 46 | Clin | Clin | 46 | 44 | 26 | 9 | 37 | 162 |
| Miso* | 22 | F | W | Yes | 34 | 9 | 43 | Clin | Clin | 13 | 46 | 2 | 13 | 35 | 109 |
| Miso* | 21 | F | W | Yes | 26 | 15 | 41 | None | Clin | 32 | 29 | 28 | 7 | 47 | 143 |
| Miso* | 19 | F | L | Yes | 34 | 12 | 46 | Clin | Clin | 48 | 22 | 5 | 2 | 50 | 127 |
| Miso* | 18 | F | W | Yes | 35 | 12 | 47 | Clin | Clin | 6 | 39 | 19 | 26 | 50 | 140 |
| Miso* | 37 | F | W | Yes | 38 | 17 | 55 | Clin | Clin | 47 | 10 | 36 | 15 | 50 | 158 |
| Miso* | 20 | NB | A | Yes | 35 | 9 | 44 | Clin | Clin | 34 | 45 | 12 | 11 | 47 | 149 |
| Miso* | 21 | F | W | Yes | 25 | 7 | 32 | Clin | Clin | 38 | 27 | 11 | 10 | 39 | 125 |
| Miso* | 26 | M | W | Yes | 35 | 8 | 43 | Clin | Clin | 23 | 19 | 1 | 13 | 37 | 93 |
| Miso* | 18 | F | B | Yes | 37 | 17 | 54 | Clin | Clin | 33 | 41 | 37 | 46 | 50 | 207 |
| Miso* | 28 | F | W | Yes | 33 | 11 | 44 | Clin | Clin | 34 | 35 | 6 | 34 | 44 | 153 |
| Miso* | 19 | F | W | Yes | 30 | 20 | 50 | Clin | Clin | 33 | 11 | 21 | 26 | 49 | 140 |
| Miso | 20 | F | W | M/S | 24 | 9 | 33 | Clin | Clin | 29 | 17 | 19 | 6 | 40 | 111 |
| Miso | 19 | F | A | M/S | 29 | 11 | 40 | Clin | Clin | 42 | 0 | 0 | 35 | 33 | 110 |
| Miso | 20 | M | L | M/S | 24 | 16 | 40 | Clin | Clin | 21 | 36 | 17 | 3 | 46 | 123 |
| Miso | 19 | F | W | No | 0 | 0 | 0 | None | None | 35 | 16 | 6 | 12 | 26 | 95 |
| Miso | 18 | F | W | M/S | 26 | 2 | 28 | Sub | Sub | 37 | 36 | 6 | 4 | 34 | 117 |
| Miso | 19 | F | A | No | 23 | 9 | 32 | None | Mild | 23 | 48 | 28 | 9 | 29 | 137 |
| Cont | 19 | M | A | M/S | 0 | 0 | 0 | None | None | 14 | 17 | 4 | 20 | 14 | 69 |
| Cont | 18 | F | B | No | 19 | 3 | 22 | None | Mild | 14 | 6 | 0 | 13 | 13 | 46 |
| Cont | 18 | F | W | M/S | 0 | 0 | 0 | None | None | 0 | 0 | 0 | 2 | 4 | 6 |
| Cont | 20 | F | A | No | 27 | 0 | 27 | Sub | Sub | 0 | 0 | 0 | 0 | 8 | 8 |
| Cont | 19 | M | A | No | 12 | 0 | 12 | None | Mild | 5 | 0 | 1 | 2 | 4 | 12 |
| Cont | 21 | F | W | M/S | 23 | 5 | 28 | None | Sub | 6 | 2 | 2 | 0 | 8 | 18 |
| Cont | 19 | M | W | Yes | 23 | 1 | 24 | None | Mild | 11 | 2 | 0 | 3 | 17 | 33 |
| Cont | 32 | F | W | M/S | 20 | 0 | 20 | None | Sub | 5 | 1 | 0 | 0 | 13 | 19 |
| Cont | 19 | M | W | No | 19 | 0 | 19 | None | Sub | 36 | 6 | 0 | 2 | 18 | 62 |
| Cont | 19 | F | W | M/S | 25 | 0 | 25 | Sub | Sub | 26 | 8 | 1 | 6 | 6 | 47 |
| Cont | 18 | F | L | Yes | 23 | 0 | 23 | None | Sub | 8 | 5 | 0 | 2 | 16 | 31 |
| Cont | 18 | F | A | No | 18 | 4 | 22 | None | Sub | 20 | 5 | 0 | 1 | 23 | 49 |
| Cont | 18 | M | W | M/S | 13 | 4 | 17 | None | None | 25 | 4 | 0 | 6 | 6 | 41 |
| Cont | 18 | F | W | No | 24 | 0 | 24 | None | Mild | 1 | 8 | 0 | 9 | 4 | 22 |
| Cont | 19 | NB | W | M/S | 14 | 6 | 20 | None | Mild | 5 | 14 | 1 | 0 | 40 | 60 |
| Cont | 19 | M | W | M/S | 19 | 2 | 21 | Sub | Sub | 11 | 7 | 0 | 7 | 1 | 26 |
| Cont | 19 | M | A | No | 14 | 2 | 16 | None | Mild | 6 | 3 | 4 | 4 | 2 | 19 |
| Cont | 20 | F | B | M/S | 10 | 0 | 10 | None | Mild | 26 | 0 | 0 | 8 | 0 | 34 |
| Cont | 19 | F | W | M/S | 0 | 0 | 0 | None | None | 5 | 0 | 0 | 0 | 2 | 7 |
| Cont* | 18 | F | B | No | 0 | 0 | 0 | None | None | 2 | 0 | 0 | 0 | 0 | 2 |
| Cont* | 18 | M | W | No | 0 | 0 | 0 | None | None | 1 | 0 | 0 | 0 | 0 | 1 |
| Cont* | 21 | M | A | No | 0 | 0 | 0 | None | None | 1 | 1 | 2 | 1 | 3 | 8 |
| Cont* | 19 | M | W | No | 0 | 0 | 0 | None | None | 1 | 0 | 0 | 0 | 0 | 1 |
| Cont* | 20 | M | W | No | 0 | 0 | 0 | None | None | 0 | 0 | 0 | 1 | 0 | 1 |
| Cont* | 20 | M | W | No | 0 | 0 | 0 | None | None | 12 | 0 | 1 | 3 | 5 | 21 |
| Cont* | 19 | M | W | No | 0 | 0 | 0 | None | None | 11 | 0 | 0 | 1 | 1 | 13 |
| Cont* | 18 | M | W | No | 5 | 0 | 5 | None | None | 20 | 0 | 0 | 7 | 3 | 30 |
| Cont* | 18 | M | W | No | 0 | 0 | 0 | None | None | 0 | 0 | 0 | 0 | 0 | 0 |
| Cont* | 20 | M | W | No | 0 | 0 | 0 | None | None | 11 | 0 | 0 | 0 | 0 | 11 |
| Cont* | 19 | O | W | No | 0 | 0 | 0 | None | None | 3 | 0 | 0 | 0 | 0 | 3 |
| Cont* | 19 | F | L | No | 0 | 0 | 0 | None | None | 6 | 0 | 0 | 0 | 0 | 6 |
| Cont* | 19 | M | A | No | 0 | 0 | 0 | None | None | 10 | 0 | 0 | 2 | 1 | 13 |
| Cont* | 18 | M | B | No | 0 | 0 | 0 | None | None | 9 | 0 | 1 | 2 | 4 | 16 |
| Cont* | 18 | F | W | No | 0 | 0 | 0 | None | None | 0 | 1 | 0 | 6 | 6 | 13 |
| Cont* | 20 | M | B | No | 0 | 0 | 0 | None | None | 4 | 0 | 0 | 0 | 4 | 8 |
| Cont* | 19 | M | W | No | 0 | 0 | 0 | None | None | 0 | 0 | 0 | 0 | 0 | 0 |
| Cont* | 20 | F | A | No | 0 | 0 | 0 | None | None | 1 | 0 | 0 | 0 | 2 | 3 |
| Cont* | 34 | F | A | No | 0 | 0 | 0 | None | None | 13 | 12 | 7 | 8 | 11 | 51 |
| Cont* | 28 | F | W | No | 0 | 0 | 0 | None | None | 17 | 0 | 0 | 10 | 7 | 34 |

**Group**: Defined by S5 (* denotes extreme group membership); Miso=misophonia, Cont=control
**Gender**: F=female, M=male, NB=nonbinary, O=other/prefer not to say
**Race:** A=Asian, B=Black, L=Latino, W=White
**Self-Report**: Response to question “Do you think you have misophonia?”; M/S=maybe/somewhat
**DVMSQ:** Duke-Vanderbilt Misophonia Screening Questionnaire
 **Symp**=symptom score (out of 40)
 **Impair**=impairment score (out of 28)
 **TOTAL**=total score (out of 68)
 **Diag**=result from diagnostic algorithm operationalised in Williams et al., 2022;

Sub=sub-clinical misophonia, Clin= clinically significant misophonia
 **Diag (adj)**=result from diagnostic algorithm, adjusted to remove Criterion D

(see Method)
**S5**: Selective Sound Sensitivity Syndrome Scale subscales (out of 50):

**Ext**=externalizing, **Int**=internalizing, **Imp**=impact, **Out**=outburst, **Thr**=threat
 **TOTAL**=total score (out of 250)

## Supplement 2: Discomfort Rating

### High vs. Low Discomfort Sounds

Across our entire sample, participants had an average of 14.8 trials (range: 0-33) included with high discomfort sounds and 47.7 trials (range: 11-96) included for low discomfort sounds; high discomfort sounds were assigned discomfort ratings of 3.3 (range: 1-5) on average, and low discomfort sounds were assigned discomfort ratings of 0.05 (range: 0-0.9) on average. Individuals with misophonia had significantly more trials of high discomfort sounds compared to controls when split by S5 groups (*t*(63) = 2.034, *p*_HB_ = 0.046), but not by Extreme groups (*t*(38) = 1.044, *p*_HB_ = 0.303). Individuals with misophonia also had significantly fewer trials of low discomfort sounds compared to controls (S5 groups: *t*(63) = -2.777, *p*_HB_ = 0.014, Extreme groups: *t*(38) = -3.375, *p*_HB_ = 0.003). For a depiction of how frequently each sound stimulus ended up in a participant’s high vs. low discomfort category, see Fig S1. Note the variation in stimulus inclusion within the same sound category, denoting a benefit of using multiple exemplars; for example, if breathing #1 was not bothersome but breathing #2 was, then only trials with breathing #2 would be analyzed as high discomfort (instead of averaging across all breathing trials).


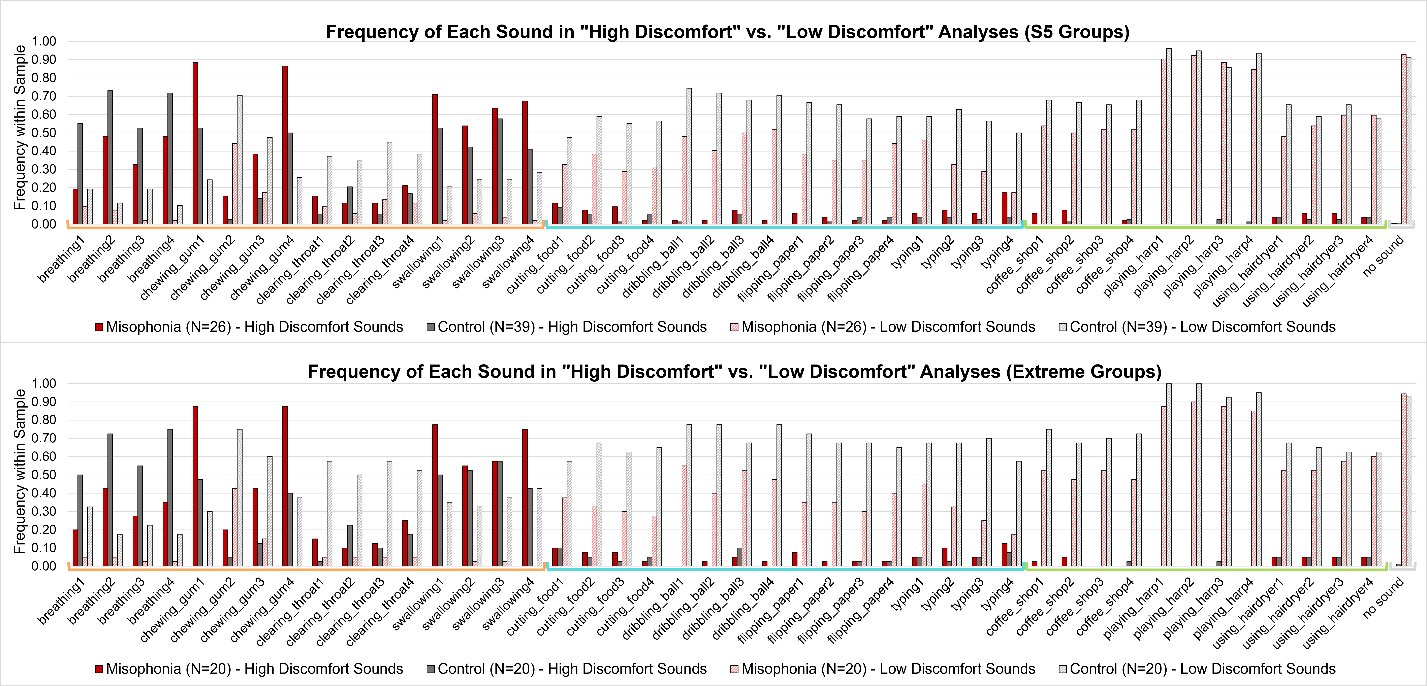


**Fig S1. Proportion of participants for which each sound stimulus was included in their “high” vs. “low” discomfort analyses.** (top) split by S5 groups, (bottom) split by extreme groups. Dark bars = high discomfort sounds, light bars = low discomfort sounds. Red = misophonia group, gray = control group. Orange = Oral sounds, blue = Other sounds, green = Control sounds, gray = Quiet.

### Discomfort Ratings Collapsed Across Groups

Across all participants, there was a significant sequential decrease of discomfort across categories: Oral sounds were rated with higher discomfort than Other sounds (*t*(64) = 13.420, *p*_HB_ < 0.001), Other sounds were rated higher than Control sounds (*t*(64) = 6.068, *p*_HB_ < 0.001), and Control sounds were rated higher than Quiet (*t*(64) = 4.556, *p*_HB_ < 0.001).


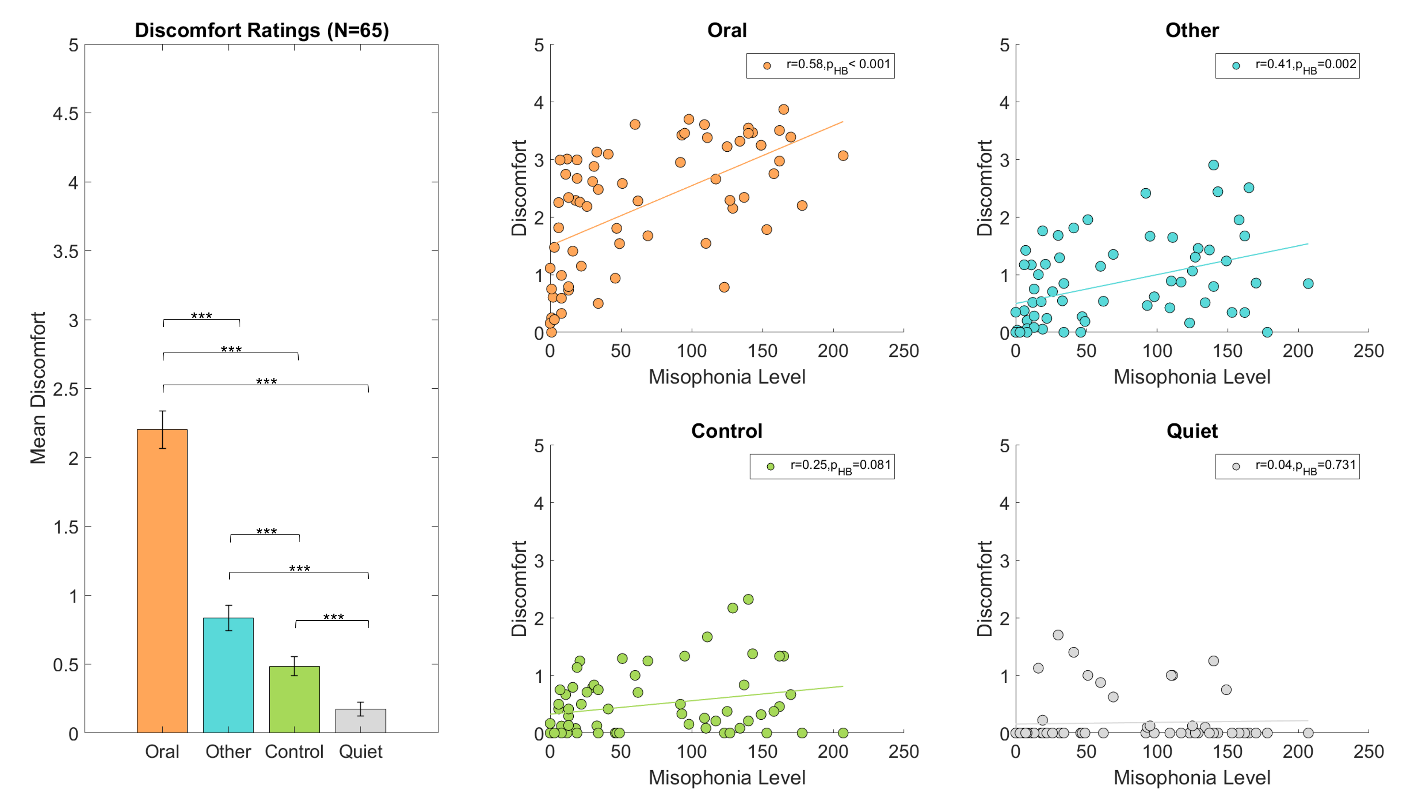


**Fig S2. Discomfort ratings by sound category**. (left) averaged across all participants, (right) scatterplots of misophonia level (S5 total score) by mean discomfort rating for each sound category. Error bars depict standard error of the mean. ***p_HB_ ≤0.001

### Discomfort Rating by Sound ID Accuracy

**Supplementary Table S*2*.** Number of participants within each comparison group in Fig 4.

|  |  | **S5 Misophonia** | **S5 Control** |
| --- | --- | --- | --- |
| **Oral** | Correct | 26 | 39 |
|  | Incorrect | 19 | 35 |
| **Other** | Correct | 26 | 39 |
|  | Incorrect | 22 | 30 |
| **Control** | Correct | 26 | 39 |
|  | Incorrect | 4 | 7 |
| **Quiet** | Correct | 26 | 39 |
|  | Incorrect | 0 | 0 |

## Supplement 3: Gender Judgment

*Median Response Times*

Using a 2 (group: misophonia vs. control, between-subjects) x 12 (sound class: breathing vs. chewing vs. clearing throat vs. swallowing vs. cutting food vs. … vs. no sound, within-subjects) mixed ANOVA, we again found significant main effects of group (*F*(1,756) = 49.444, *p* < 0.001, η_p_^2^ = 0.061) and class (*F*(11,756) = 7.543, *p* < 0.001, η_p_^2^ = 0.099). Evident most clearly in the analysis with extreme groups, pre-planned independent samples *t*-tests revealed that individuals with misophonia were significantly slower than controls at responding to the gender judgment during certain classes: clearing throat (Misophonia: *M* = 2.26s, *SD* = 1.35s; Control: *M* = 1.43s, *SD* = 0.40s; *t*(42) = 2.934, *p*_HB_ = 0.049, Hedges’ *g* = 0.883), flipping paper (Misophonia: *M* = 2.577s, *SD* = 1.64s; Control: *M* = 1.48s, *SD* = 0.62s; *t*(42) = 3.128, *p*_HB_ = 0.038, Hedges’ *g* = 0.942), typing (Misophonia: *M* = 2.98s, *SD* = 1.77s; Control: *M* = 1.76s, *SD* = 0.89s; *t*(42) = 3.005, *p*_HB_ = 0.045, Hedges’ *g* = 0.905), and no sound (Misophonia: *M* = 3.26s, *SD* = 1.76s; Control: *M* = 1.91s, *SD* = 1.13s; *t*(42) = 3.107, *p*_HB_ = 0.038, Hedges’ *g* = 0.936).

**Fig S3. Gender judgment median response time by sound category, averaged across samples**. Top row: split by S5 groups across (left) sound category and (right) sound class. Bottom row: split by extreme groups across (left) sound category and (right) sound class. bars depict standard error of the mean. *p_HB_ ≤0.05


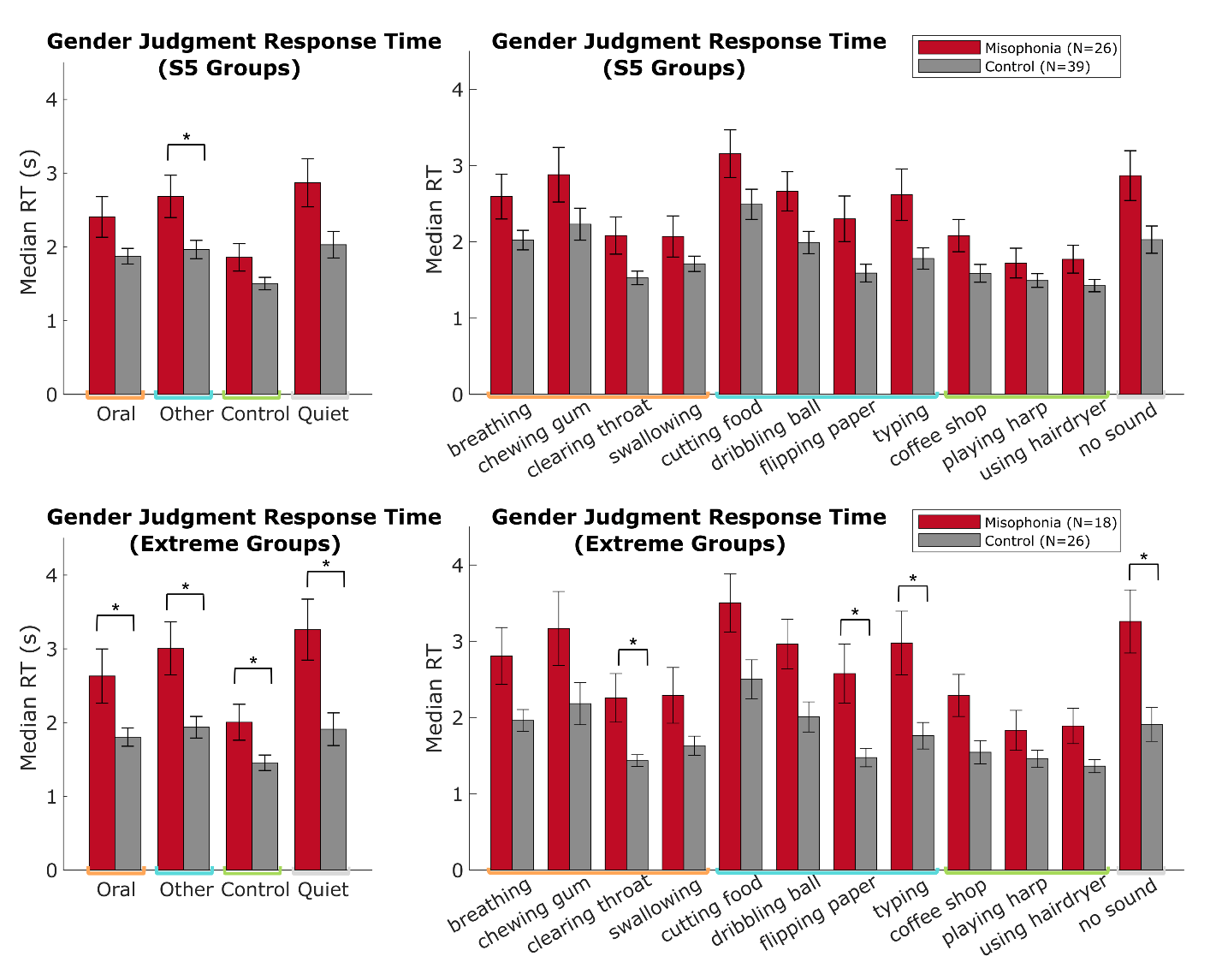


### Gender Judgment Performance Collapsed Across Groups

Collapsed across both groups, accuracy on the gender judgment was significantly lower while performed during an Oral sound compared to all other categories (Other: t(64) = -3.200, p_HB_ = 0.006; Control: t(64) = -3.642, p_HB_ = 0.003; Quiet: t(64) = -5.049, p_HB_ < 0.001). Accuracy was also significantly lower during an Other sound compared to Quiet (t(64) = -3.663, p_HB_ = 0.003). However, accuracy was not generally impaired by any acoustic stimuli, as there was no
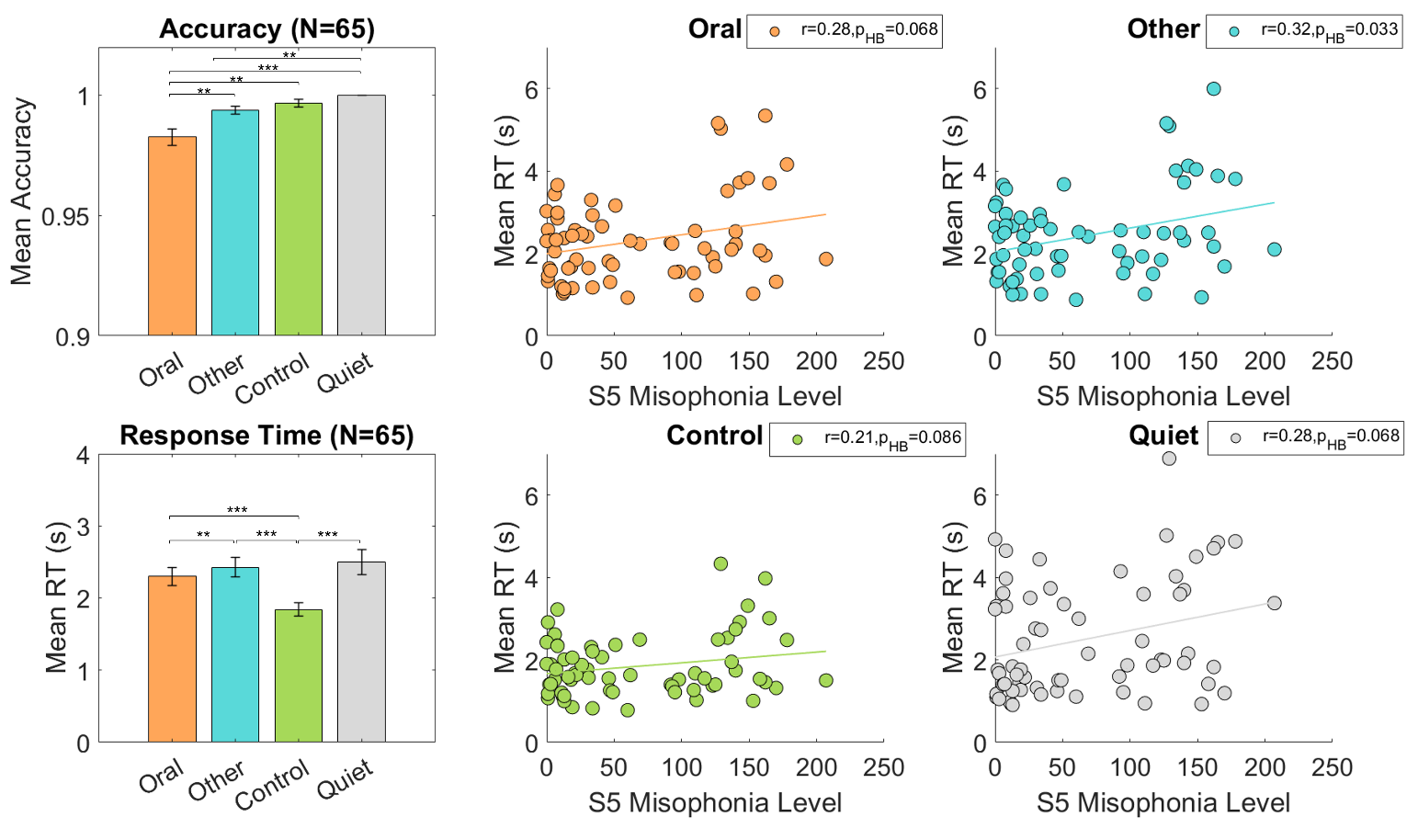
significant difference between Control sounds and Quiet (t(64) = -1.929, p_HB_ = 0.116).

**Fig S4. Gender judgment performance by sound category**. (top left) accuracy averaged across all participants, (bottom left) mean response time averaged across all participants, (right) scatterplots of misophonia level (S5 total score) by mean response time for each sound category. Error bars depict standard error of the mean. **p_HB_ ≤0.01, ***p_HB_ ≤0.001

Participants were overall fastest during trials with Control sounds compared to all other categories (Oral: *t*(64) = 7.953, *p*_HB_ = 1.954 x 10^-10^, Other: *t*(64) = 9.257, *p*_HB_ = 1.209 x 10^-12^, Quiet: *t*(64) = -5.988, *p*_HB_ = 4.244 x 10^-7^); this pattern was also found in each group separately.

## Supplement 4: Likeability Rating

###
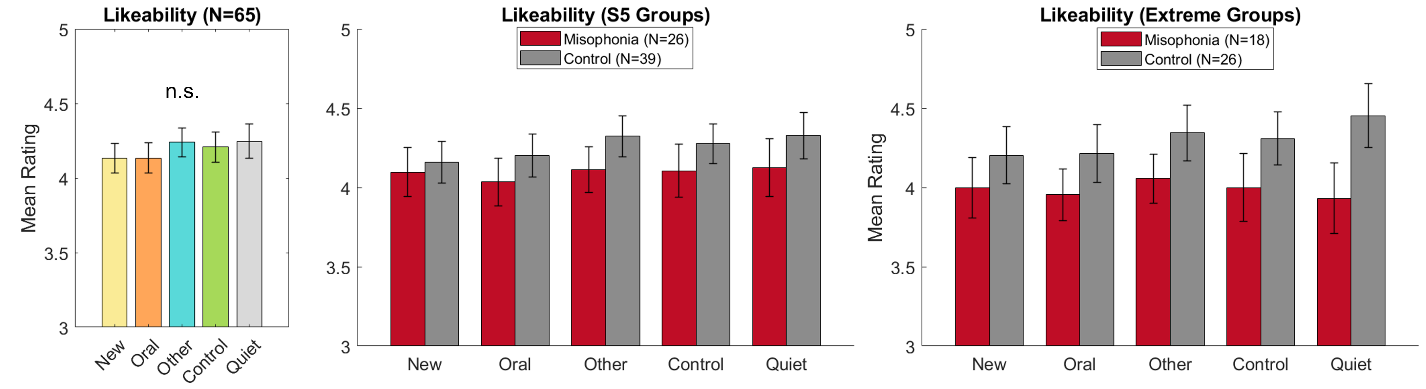


**Fig S5. Likeability ratings by sound category.** (left) collapsed across all participants, (middle) split by S5 groups, (right) split by extreme groups. Error bars depict standard error of the mean.

### Likeability Ratings with Different High vs. Low Discomfort Thresholds

Using samples split by S5 groups and a conservative (10%) threshold for determining a participant’s high vs. low discomfort sounds, both misophonic individuals and controls rate faces paired with high discomfort sound memories as significantly less likeable than faces paired with low discomfort sound memories (misophonia: *t*(25) = -5.434, *p*_HB_ = 1.043 x 10^-4^; control: *t*(33) = -3.385, *p*_HB_ = 0.013) (Fig S6). However, when using a more liberal (25%) threshold, only misophonic individuals show this pattern (*t*(25) = -5.278, *p*_HB_ = 1.638 x 10^-4^); controls show no difference in likeable ratings based on the discomfort of the sound reported. Thus, it seems when individuals view a face they don’t like, they’re internally attributing the dislike to the face’s association with a bothersome sound; misophonic individuals just have a lower threshold for sound aversion, since their likeable ratings differ even for a liberal discomfort cutoff.


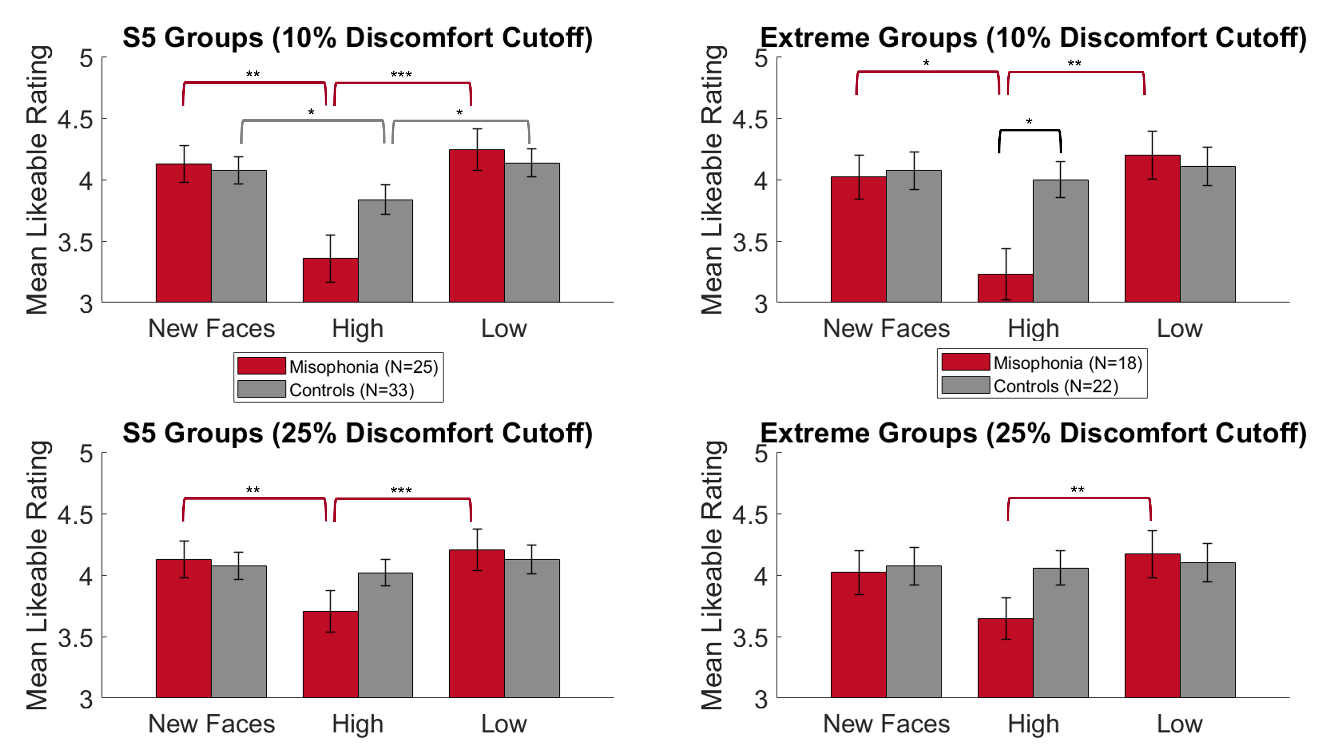


**Fig S6. Likeable ratings by subjective sound discomfort, split by cutoff threshold.** (top) 10% cutoff, reproduced from Fig 6E-F. (bottom) 25% cutoff. (left) split by S5 groups, (right) split by extreme groups. †p_HB_≤0.10, *p_HB_ ≤0.05, **p_HB_ ≤0.01, ***p_HB_ ≤0.001

### Likeability Rating by Sound Memory Accuracy

**Supplementary Table S3.** Number of participants within each comparison group across Fig 7A.

|  |  | **S5 Misophonia** | **S5 Control** |
| --- | --- | --- | --- |
| **Oral** | Correct | 20 | 23 |
|  | Incorrect | 25 | 33 |
| **Other** | Correct | 24 | 28 |
|  | Incorrect | 25 | 33 |
| **Control** | Correct | 20 | 26 |
|  | Incorrect | 25 | 33 |
| **Quiet** | Correct | 13 | 18 |
|  | Incorrect | 25 | 31 |

**Supplementary Table S4.** Number of participants within each comparison group across Fig 7B.

|  |  | **S5 Misophonia** | **S5 Control** |
| --- | --- | --- | --- |
| **breathing** | Correct | 10 | 13 |
|  | Incorrect | 25 | 32 |
| **chewing gum** | Correct | 11 | 10 |
|  | Incorrect | 25 | 33 |
| **clearing throat** | Correct | 9 | 11 |
|  | Incorrect | 25 | 33 |
| **swallowing** | Correct | 2 | 6 |
|  | Incorrect | 25 | 33 |
| **cutting food** | Correct | 13 | 14 |
|  | Incorrect | 25 | 33 |
| **dribbling ball** | Correct | 16 | 21 |
|  | Incorrect | 25 | 33 |
| **flipping paper** | Correct | 12 | 11 |
|  | Incorrect | 25 | 33 |
| **typing** | Correct | 11 | 18 |
|  | Incorrect | 25 | 33 |
| **coffee shop** | Correct | 11 | 15 |
|  | Incorrect | 25 | 33 |
| **playing harp** | Correct | 15 | 15 |
|  | Incorrect | 25 | 33 |
| **using hairdryer** | Correct | 14 | 19 |
|  | Incorrect | 24 | 32 |
| **no sound** | Correct | 13 | 18 |
|  | Incorrect | 25 | 31 |

**Supplementary Table S5.** Number of participants within each comparison group across Fig 7C.

|  |  | **S5 Misophonia** | **S5 Control** |
| --- | --- | --- | --- |
| **New Faces** | Correct | 25 | 33 |
|  | Incorrect | 24 | 33 |
| **High Discomfort** | Correct | 16 | 17 |
|  | Incorrect | 25 | 32 |
| **Low Discomfort** | Correct | 24 | 32 |
|  | Incorrect | 25 | 33 |

Supplement 5: Memory Performance

Pre-planned t-tests revealed lower overall memory for faces paired with Oral sounds compared to both Control sounds (*t*(64) = -3.334, *p*_HB_ = 0.007) and Quiet (*t*(64) = -3.788, *p*_HB_ = 0.002), and marginally worse memory for faces paired with Other sounds compared to Control sounds (*t*(64) = -2.196, *p*_HB_ = 0.095) and Quiet (*t*(64) = -2.507, *p*_HB_ = 0.059). Face memory did not significantly vary by misophonia level for any category.


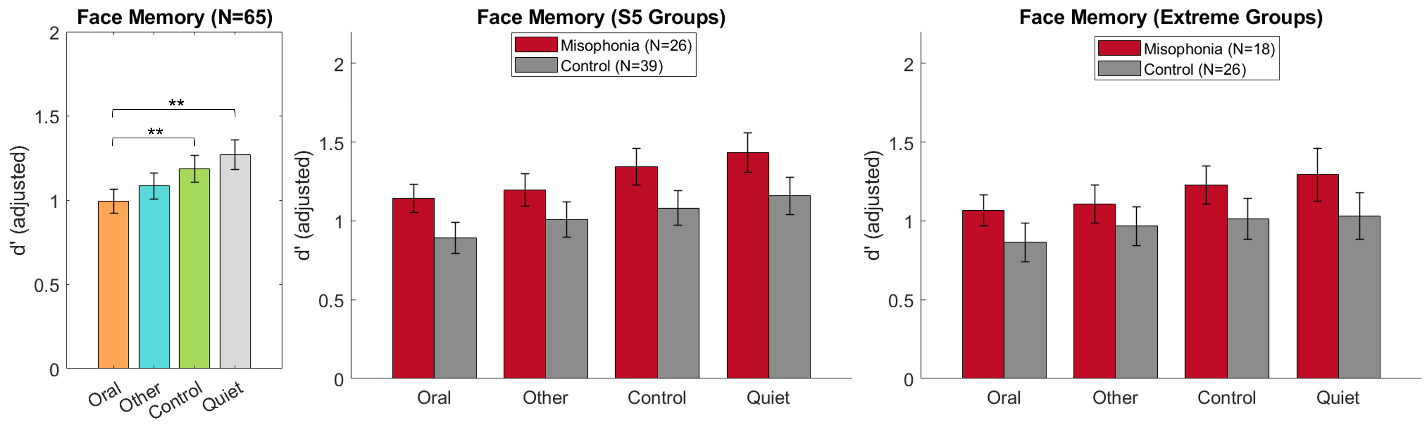


**Fig S7. Face memory performance by sound category.** (left) collapsed across all participants, (middle) split by S5 groups, (right) split by extreme groups. Error bars depict standard error of the mean. **p_HB_ ≤0.01
